# APP Induces AICD‐Mediated Autophagy‐Dependent Axon Degeneration

**DOI:** 10.1111/acel.70301

**Published:** 2025-11-23

**Authors:** Jingjing Luo, Yu Qiu, Yu Pan, Ruihong Xu, Yi Sun, Yihao Sun, Luming Zhuang, Elleen Xue, Wenzhe Li, Qian Zhou, Zhongwei Lv, Chenglin Li, Lei Xue

**Affiliations:** ^1^ Department of Nuclear Medicine, Shanghai 10th People's Hospital, Shanghai Key Laboratory of Signaling and Diseases Research, School of Life Science and Technology Tongji University Shanghai China; ^2^ Mathey College Princeton University Princeton New Jersey USA; ^3^ The First Rehabilitation Hospital of Shanghai, School of Medicine Tongji University Shanghai China

**Keywords:** AICD, Alzheimer's disease, APP, autophagy, axon degeneration

## Abstract

The amyloid precursor protein (APP) plays a pivotal role in the pathogenesis of Alzheimer's disease (AD). While the production of Amyloid beta (Aβ) has traditionally been considered the primary cause of AD, the role of the APP intracellular domain (AICD) remains largely elusive. In this study, we established a novel model in the adult fly wing by expressing human APP, recapitulating AD‐associated axon degeneration. Using this model, we discovered that ectopic APP expression in *Drosophila* wing margin neurons led to age‐dependent axon degeneration. APP's effect depended on AICD production, and AICD overexpression alone was sufficient to induce axon degeneration in adult wings. Further investigations indicated that APP‐ or AICD‐induced axon degeneration could be alleviated by blocking autophagy, but not apoptosis. Additionally, we identified a FoxO/Snail–Atg1 axis as an essential mediator of APP/AICD‐induced autophagy‐dependent axon degeneration. Finally, we demonstrated that administration of chloroquine, an autophagy inhibitor, effectively ameliorates APP‐ or AICD‐induced axon degeneration. Our findings provide crucial insights into how APP induces autophagy‐dependent axon degeneration through AICD production, laying a foundation for future investigations into AD pathogenesis.

## Graphical abstract

In the adult Drosophila wing, amyloid precursor protein (APP) promotes axon degeneration through its intracellular domain (AICD) via an autophagy‐dependent pathway. The transcription factor dFoxO is essential for APP/AICD‐induced autophagy and plays a critical role in mediating the resulting neurodegeneration.

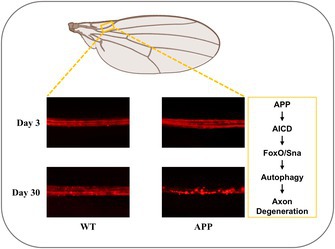

## 1 Introduction

Alzheimer's disease (AD) is an age‐associated degenerative disorder originating from lesions or degeneration within the central nervous system (Querfurth and LaFerla 2010; Wood et al. 2020). Current estimates indicate that AD affects over 35 million individuals worldwide (Deng et al. 2020). Key pathological features of AD include the presence of extracellular senile plaques, intracellular neurofibrillary tangles, and neuronal dysfunction (Goedert et al. 1991; Lammich et al. 1999; Torroja, Chu, et al. 1999). The amyloid precursor protein (APP) is a critical player in the pathophysiology of AD (Chow et al. 2010). APP can undergo multiple cleavages to yield various fragments, including N‐APP, Aβ peptides, and the APP intracellular domain (AICD) (Koo et al. 1993; Sinha and Lieberburg 1999; O'Brien and Wong 2011). Although the exact mechanisms underlying AD remain debated, the Aβ hypothesis has emerged as a leading explanation for its pathogenesis (Herrup 2010). Consequently, numerous research efforts have focused on reducing Aβ levels in AD patients, with limited success in clinical trials (Egan et al. 2019). Furthermore, evidence suggests that synaptic dysfunction precedes plaque formation in certain AD models, implying that Aβ plaques may play a role in later AD stages (Oddo et al. 2003). As a result, attention has shifted toward understanding the role of AICD in AD pathogenesis. Ectopic expression of APP has been shown to induce AICD‐dependent cell death (Wang et al. 2014), and AICD has been reported to form a complex with Fe65 and Tip60 to regulate target gene expression (Cao et al. Cao and Sudhof 2001; Baek et al. 2002).

*Drosophila melanogaster* has proven to be an excellent model for studying the pathological features of AD, with various transgenic models expressing Aβ or APP driven by robust exogenous promoters in larvae and adult flies (Fang et al. 2012; Guo et al. 2012; Wang et al. 2014; Peng et al. 2015). The *Appl* gene encodes the *Drosophila* homolog of APP (Luo et al. 1992), and prior research has highlighted the crucial role of Appl in axon transport (Torroja, Packard, et al. 1999; Gunawardena and Goldstein 2001). While impaired axonal transport is a significant contributor to pathological changes in AD (Christensen et al. 2014), the precise mechanisms leading to axonopathy remain elusive.

In this study, we established an in vivo model of axon degeneration using the adult fly wing. Our investigation revealed that ectopic expression of APP in *Drosophila* wing neurons induced AICD‐dependent axon degeneration. Additionally, our observations indicated that axon degeneration triggered by APP or AICD can be effectively mitigated by inhibiting autophagy, but not apoptosis. Furthermore, our research identified that FoxO and Snail are critical mediators in APP/AICD‐induced autophagy and subsequent axon degeneration. Moreover, Atg1 is both necessary and sufficient for APP/AICD‐induced axon degeneration. Importantly, we showed that treatment with the autophagy inhibitor chloroquine effectively alleviates axon degeneration induced by APP or AICD. These findings offer valuable insights into the role of AICD in Alzheimer's disease pathology, enhancing our mechanistic understanding of the disease and highlighting a pathway that warrants further exploration to assess its therapeutic potential.

## 2 Results

### 2.1 APP Induces Age‐Dependent Axon Degeneration in Wing Margin Neurons

**FIGURE 1:**
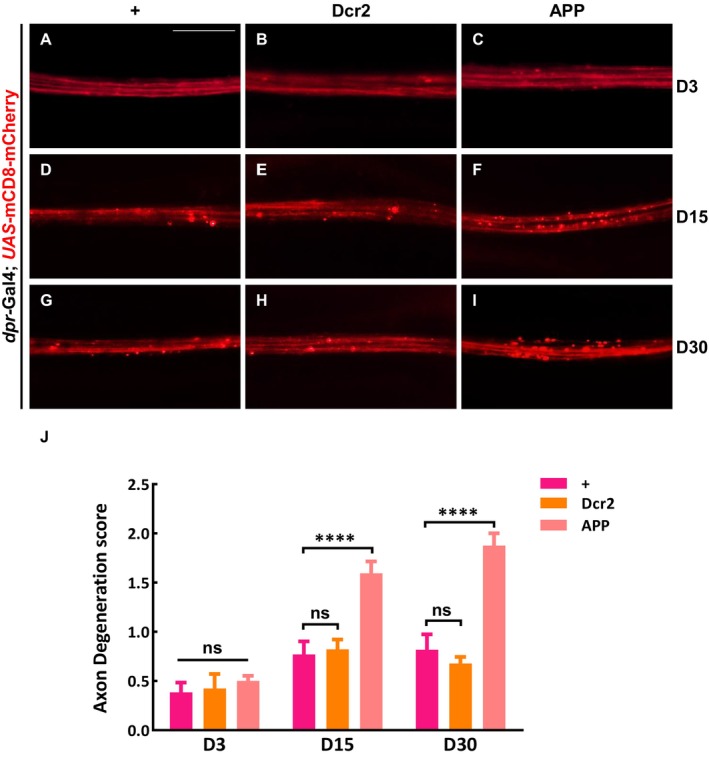
APP induces age‐dependent axon degeneration in *Drosophila* wing axons. (A–C) Depiction of the wing arch in 3‐day‐old adult females. The axons of the control group (A), the negative control with Dcr2 expression (B), and the APP over‐expressed group (C) appeared smooth and fibrous; (D–F) The wing arch of 15‐day‐old adult females is shown. Compared with the control group (D, E), overexpression of APP induced a large number of beaded spots and fragment in the axon (F); (G–I) Schematic diagram of 30‐day‐old *Drosophila* axon. The axon in the control group remained continuous and smooth, with only a few beads (G, H), while overexpression of APP resulted in severe fragmentation, aggregated plaque, and complete destruction of axon integrity (I); (J) Statistical analysis of axon degeneration in different genotypes (*n* > 15 per phenotype). Values are presented as mean ± SEM. ns represents not significant, and **** represents *p* < 0.0001. Scale bar: 20 μm.

To visualize the nervous system in living animals, we expressed mCD8‐mCherry in chemosensory neurons along the anterior wing margin driven by *dpr*‐Gal4 (Figure S1A) (Fang et al. 2012). We took advantage of this system to express human APP (hAPP695), and characterized its influence on axon integrity based on an evaluation system that measures the severity of axon degeneration (Figure S1B–E) (Fang et al. 2012). Expression of Dcr2 was included as a negative control to demonstrate that the APP‐induced phenotype is not simply a result of random protein overexpression. We found that at 3 days (D3) post‐eclosion, APP‐expressing flies exhibited a smooth, fiber‐like mCherry signal, similar to the two control groups (Figure 1A–C,J). By contrast, at 15 days (D15), while control flies showed some bead‐like dots and fragments on their axons, APP‐expressing flies displayed significantly more severe phenotypes (Figure 1D–F,J). By day 30 (D30), ectopic APP expression not only led to the presence of numerous bead‐like dots and fragments on the axons but also caused severe disruption of axon integrity compared with age‐matched controls (Figure 1G–J). These results indicate that APP overexpression triggers age‐dependent axon degeneration in adult wing margin neurons, resembling the effects of axon injury.

### 2.2 APP Induces Axon Degeneration Through AICD

**FIGURE 2:**
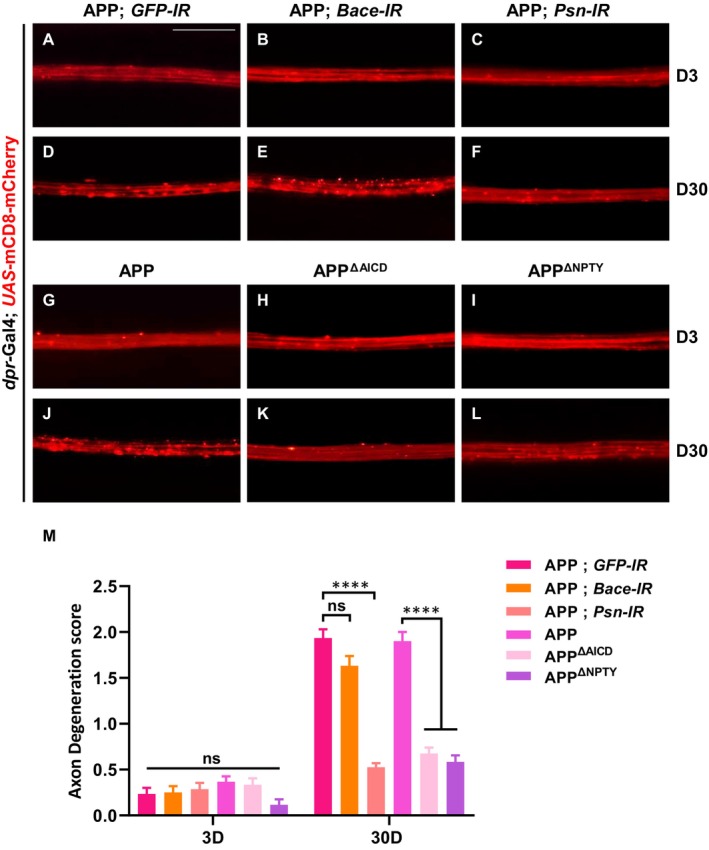
APP induces axon degeneration through AICD. (A–L) The wing arch of adult females is shown. No significant axon degeneration was observed in 3‐day‐old wing arch of each genotype (A–C, G–I). On day 30, compared with APP‐expressing group (D), down‐regulation of *BACE* had no significant effect on APP‐induced axonal degeneration (E), while down‐regulation of *Psn* significantly suppressed APP‐induced axon degeneration on day 30 (F). Compared with the control group (J), expression of APP^ΔAICD^ or APP^ΔNPTY^ did not exhibit an obvious axon degeneration phenotype (K, L); Statistical analysis of axon degeneration in different genotypes (*n* > 15 per phenotype) (M). Values are shown as mean ± SEM, ns represents not significant, and **** represents *p* < 0.0001. Scale bar: 20 μm.

APP is cleaved by β‐ and γ‐secretases, resulting in the production of Aβ and the APP intracellular domain (AICD) (Baek et al. 2002; Chow et al. 2010). To determine which fragment contributes to APP‐induced axon degeneration, we first depleted β‐secretase (BACE), an enzyme necessary for Aβ production (Qahwash et al. 2004). We observed that knocking down *BACE* did not affect APP‐induced axon degeneration on day 30 (Figure 2D,E,M), suggesting that Aβ may not be involved in this process. In contrast, RNAi‐mediated depletion of *Presenilin* (*Psn*), a critical component of the γ‐secretase complex responsible for cleaving AICD from APP, effectively inhibited APP‐induced axon degeneration (Figure 2F,M). These findings suggest that APP‐induced axon degeneration is mediated by AICD. Furthermore, the expression of truncated APP proteins lacking either AICD (APP^ΔAICD^) or the NPTY motif in AICD (APP^ΔNPTY^) failed to promote axon degeneration on day 30 (Figure 2J–M), despite being expressed at levels comparable to full‐length APP (Figure S2). Besides, there was no significant difference between each group on day 3 (Figure 2A–C,G–I,M). These results highlight the essential role of AICD, particularly the NPTY motif, in APP‐induced axon degeneration.

**FIGURE 3:**
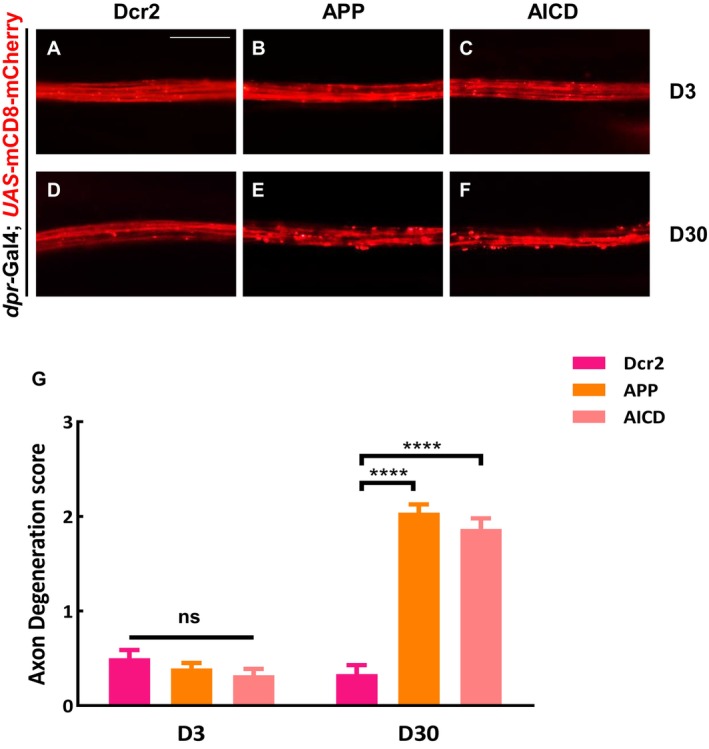
AICD Overexpression induces axon degeneration. (A–F) The wing arch of adult females is shown. The axons of different genotypes appeared smooth and continuous on day 3 (A–C). Compared with the control (D), ectopic expression of APP (E) or AICD (F) triggered axon degeneration on day 30. Statistical analysis of axon degeneration in different genotypes (*n* > 15 per phenotype) (G). Values are presented as mean ± SEM, ns represents not significant, and **** represents *p* < 0.0001. Scale bar: 20 μm.

To investigate whether AICD alone is sufficient to induce axon degeneration, we generated a *UAS*‐AICD transgene that included the signal peptide and transmembrane domain of APP to mimic its membrane localization. Intriguingly, the expression of AICD was sufficient to trigger axon degeneration (Figure 3). Overexpression of APP or AICD did not affect the protein levels of mCD8‐mCherry (Figure S3). To further validate our observation, we used two additional markers: the membrane‐targeted mCD4‐GFP and the microtubule‐bound EB1‐GFP. Consistently, ectopic expression of APP or AICD resulted in a similar axon degeneration phenotype by day 30 (Figure S4). These results confirm that AICD is both necessary and sufficient for APP‐induced axon degeneration.

### 2.3 Apoptosis Is Not Involved in APP‐Induced Axon Degeneration

Axon degeneration is a fundamental process in neuronal development, plasticity, injury response, and neurodegenerative diseases (Wang et al. 2012; Geden and Deshmukh 2016). Previous studies have suggested that the Death Receptor 6 (DR6) mediates APP‐induced neuronal cell body death and axonal pruning through caspase‐dependent mechanisms (Nikolaev et al. 2009), and apoptosis‐induced axon degeneration is known to occur in response to various neuronal insults (Lu et al. 2017; Woolums et al. 2020). To determine whether APP‐induced axon degeneration depends on caspase‐mediated apoptosis, we inhibited the apoptotic cascade by expressing the baculoviral anti‐apoptotic protein P35 or the *Drosophila* inhibitor of apoptosis DIAP1, or by knocking down the effector caspase *Dcp1*. However, inhibiting apoptosis failed to suppress APP‐induced axon destruction (Figure S5), suggesting that apoptosis is not involved in APP‐induced axon degeneration.

### 2.4 APP/AICD Promotes Autophagy‐Mediated Axon Degeneration

Aberrant autophagy has been detected in postmortem brain samples from AD patients (Yu et al. 2004; Nixon 2007) and has been shown to contribute to APP‐induced pathological functions in vivo (Zhuang et al. 2020). To investigate the potential role of autophagy in APP‐triggered axon degeneration, we examined autophagy by expressing mCherry‐Atg8a, a widely used autophagosome marker. Compared with controls, flies expressing APP or AICD displayed an increased number of mCherry‐Atg8a puncta in D30 wing margin axons (Figure S6). Additionally, an increased number of mCherry‐Atg8a puncta was observed in third‐instar larval peripheral nerves upon expressing APP or AICD driven by *Appl*‐Gal4 (Figure S7). Furthermore, we employed LAMP‐GFP and LysoTracker to label autolysosomes, thereby providing additional confirmation of autophagy occurrence. Compared with controls, ectopic expression of APP or AICD increased the number of autolysosomes in wing margin axons and LysoTracker‐positive puncta in the peripheral nerves of third‐instar larvae (Figure S8). These findings indicate that APP or AICD expression is sufficient to activate autophagy in axons.

**FIGURE 4:**
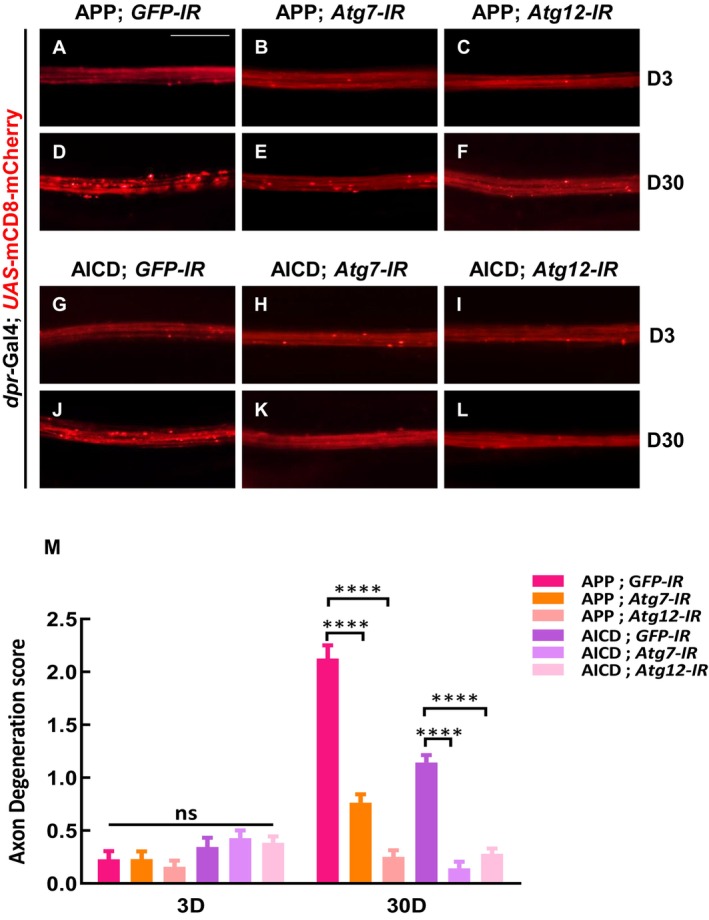
Inhibition of autophagy impedes APP‐induced axon degeneration. (A–L) The wing arch of adult females is shown. The axons of different genotypes appeard flat, smooth and continuous on day 3 (A–C, G–I). Knockdown of *Atg7* (E) or *Atg12* (F) significantly suppressed APP‐induced axonal degeneration at day 30 when compared with the control (D). Compared with the control group (J), depletion of *Atg7* (K) or *Atg12* (L) significantly inhibited AICD‐induced axon degeneration on day 30. Statistical analysis of axon degeneration in different genotypes (*n* > 15 per phenotype) (M). Values are shown as mean ± SEM. ns represents not significant, and **** represents *p* < 0.0001. Scale bar: 20 μm.

To explore whether enhanced autophagy contributes to APP/AICD‐induced axon degeneration, we blocked autophagy by depleting *Atg7* or *Atg12*, both essential for autophagosome assembly. Knockdown of either *Atg7* or *Atg12* significantly suppressed APP or AICD‐induced axon degeneration (Figure 4), while ectopic expression of *Atg7‐IR* or *Atg12‐IR* alone had no significant effect on wing margin axons (Figure S9), suggesting that APP/AICD‐induced axon degeneration is mediated by autophagy.

### 2.5 Inhibition of Autophagy by Chloroquine Impedes APP/AICD‐Induced Axon Degeneration

**FIGURE 5:**
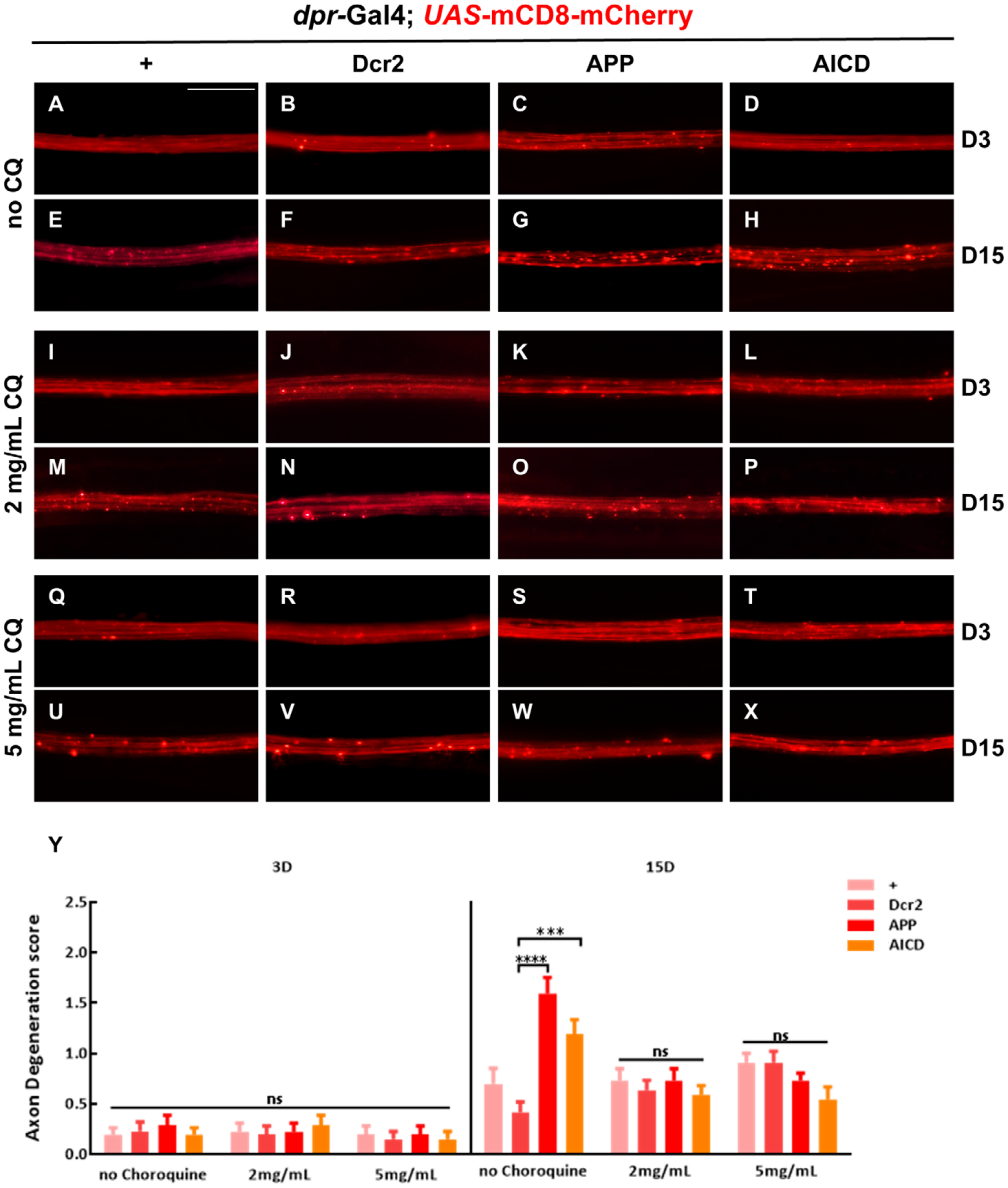
Inhibition of autophagy by chloroquine impedes APP‐induced axon degeneration. (A–X) The wing arch of adult females is shown. The axons of different genotypes were smooth and continuous on day 3 without (A–D) or with (I–L, Q–T) the treatment of chloroquine. Treatment with chloroquine at concentration of 2 mg/mL (O, P) or 5 mg/mL (W, X) strongly inhibited axon degeneration induced by ectopic expression of APP (G) or AICD (H) on day 15. Statistical analysis of axon degeneration in different genotypes (*n* > 15 per phenotype) (Y). Values are shown as mean ± SEM, ns represents not significant, *** represents *p* < 0.001, and **** represents *p* < 0.0001. Scale bar: 20 μm.

To further verify the role of autophagy in APP/AICD‐induced axon degeneration, we treated flies expressing APP or AICD with chloroquine (CQ), a well‐known autophagy inhibitor (Pasquier 2016). Based on previous studies (Nagy et al. 2013; Zirin et al. 2013; Campos‐Blazquez et al. 2022) and our preliminary experiments, we selected CQ concentrations of 2 mg/mL or 5 mg/mL. Flies raised on standard media supplemented with CQ at either concentration exhibited a significant reduction in APP or AICD‐induced axon degeneration compared with age‐matched controls (Figure 5). To rule out the possibility that CQ might affect the expression of mCD8‐mcherry, we measured the protein levels of mCD8‐mcherry across different CQ concentrations and found no changes (Figure S10). These data imply that inhibition of autophagy by chloroquine ameliorates APP‐ or AICD‐induced axon degeneration.

### 2.6 APP‐Induced Axon Degeneration Depends on dFoxO

**FIGURE 6:**
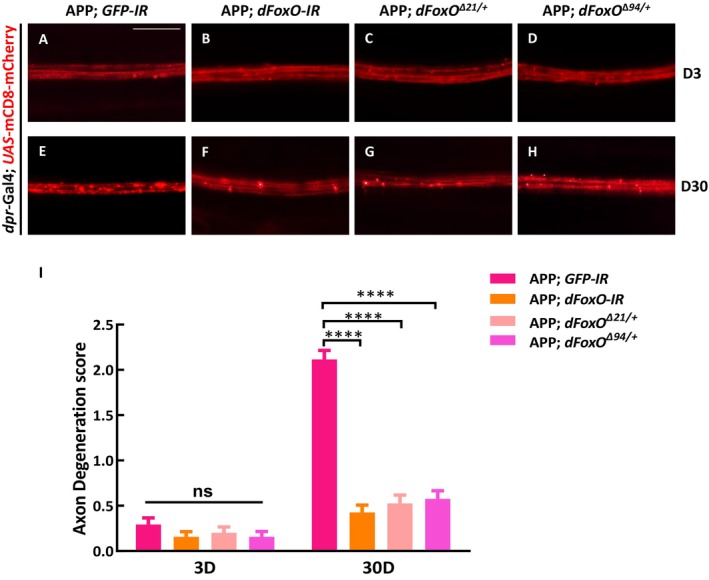
APP‐induced axon degeneration depends on dFoxO. (A–H) The wing arch of adult females is shown. The axons of different genotypes were smooth and continuous on day 3 (A–D). Ectopic APP‐induced axonal degeneration on day 30 (E) was significantly suppressed by depletion of *dFoxO* (F), or in heterozygous *dFoxO* ^*∆21*^/+ or *dFoxO* ^*∆94*^/+ background (G, H). Statistical analysis of axon degeneration in different genotypes (*n* > 15 per phenotype) (I). Values are shown as mean ± SEM, ns represents not significant, and **** represents *p* < 0.0001. Scale bar: 20 μm.

**FIGURE 7:**
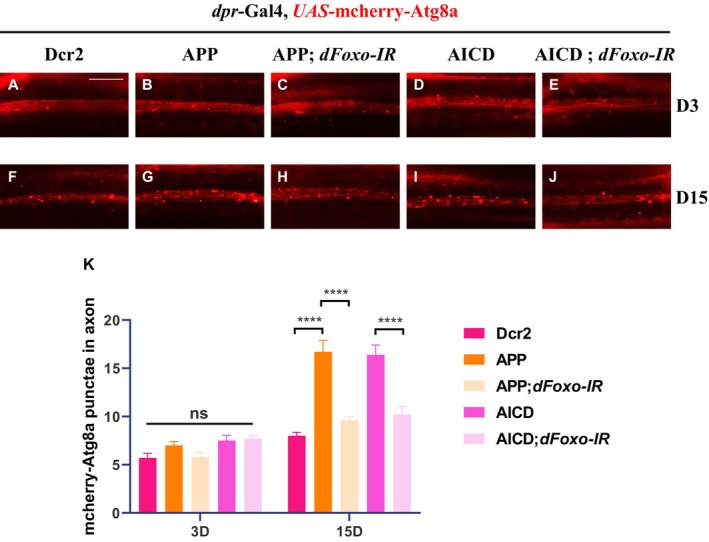
Depletion of *dFoxO* inhibits APP or AICD induced autophagosome accumulation in axons. (A–J) Depiction of the wing arch in adult flies. Driven by *dpr*‐Gal4, a similar number of mCherry‐Atg8a puncta was observed across all genotypes at day 3 (A–E). By day 30, compared with the control group (F), an increased number of mCherry‐Atg8a puncta was observed upon expression of APP (G) or AICD (I), which was suppressed by knockdown of *dFoxO* (H, J). (K) Statistical analysis of mCherry‐Atg8a puncta numbers across different genotypes (*n* = 10 per phenotype). Values are expressed as mean ± SEM, ns denotes not significant, and **** represents *p* < 0.0001. Scale bar: 20 μm.

To elucidate the mechanism by which APP triggers autophagy‐mediated axon degeneration, we hypothesized that the transcription factor FoxO might be a potential downstream mediator, as FoxO family proteins are well‐known autophagy initiators (Guo et al. 2022), and have been implicated in APP‐induced cell death (Wang et al. 2014). Intriguingly, FOXO exhibits a dual role in the context of neurodegeneration and neuroprotection. The activation of FOXO has been documented to induce cell death in mammalian neurons (Yuan et al. 2009), whereas a constantly active FOXO confers protection to neurons against proteotoxic and excitotoxic challenges (Mojsilovic‐Petrovic et al. 2009). We first examined whether *Drosophila* FoxO (dFoxO) is required for APP‐induced axon degeneration. Remarkably, *RNAi*‐mediated depletion of *dFoxO*, as well as heterozygous mutations in *dFoxO* ^*∆21*^ or *dFoxO* ^*∆94*^, significantly suppressed APP‐induced axon degeneration at day 30 (Figure 6E–I). Consistently, the increased number of mCherry‐Atg8a puncta in wing margin axons observed upon APP or AICD overexpression was also suppressed by *dFoxo* knockdown (Figure 7). A previous study showed that FoxO may regulate autophagy via its interaction with Snail (Guo et al. 2022). To further reveal that the function of FoxO in regulating autophagy participates in APP‐induced axon degeneration, we tested whether Snail is similarly involved in the aforementioned process. Indeed, knockdown of *snail* inhibited both APP‐ or AICD‐triggered autophagy and axon degeneration (Figure S11). These results indicate that dFoxO‐mediated regulation of autophagy, likely through its interaction with Snail, is required for APP‐induced autophagy‐dependent axon degeneration.

To test whether *dFoxO* suffices to trigger axon degeneration, we achieved dFoxO overexpression with *UAS*‐dFoxO (Puig et al. 2003) or *dFoxO* ^*EY11248*^, a UAS‐bearing EP element inserted in the *dFoxO* promoter (Zhao et al. 2008). Compared with age‐matched controls, overexpression of dFoxO led to bead‐like dots and fragmentations along axons at day 30 (Figure S12), suggesting that ectopic dFoxO expression is sufficient to induce axon degeneration in *Drosophila* wing margin neurons. Furthermore, our results showed that overexpression of AICD upregulated the expression of *dFoxo* (Figure S13A) and its downstream target gene *Atg1* (Figure S13B). Consistently, AICD‐induced *Atg1* expression was suppressed by *RNAi*‐mediated depletion of *dFoxO* (Figure S13B). Furthermore, knockdown of *Atg1* significantly attenuated APP‐ or AICD‐induced axon degeneration (Figure S14A–I). Importantly, overexpression of Atg1 alone was sufficient to induce axon degeneration in wing margin neurons (Figure S14J–N). Together, these results suggest that the dFoxO/Snail‐Atg1 axis is a crucial downstream pathway that mediates APP/AICD‐induced autophagy‐dependent axon degeneration.

## 3 Discussion

Despite ongoing debates regarding the detailed molecular mechanisms of AD, the disease remains a prevalent neurodegenerative condition worldwide. Impairments and abnormalities in axonal transport have been reported in various neurodegenerative diseases (Hao et al. 2019) and are strongly implicated in the pathological changes observed in AD (Xu et al. 2014). In this study, we established a novel AD model in the adult fly wing by expressing human APP, recapitulating the axon degeneration features seen in AD. This model could contribute to identifying and characterizing the degenerative processes induced by ectopic APP expression. Our findings revealed that ectopic expression of APP triggered age‐dependent axon degeneration in *Drosophila* wings, and this effect was dependent on the production of AICD. Interestingly, ectopically expressed AICD is also localized to the axonal region, where it forms aggregates in an age‐dependent manner (Figure S15). Furthermore, the axon degeneration induced by APP or AICD could be suppressed by inhibiting autophagy, but not apoptosis. We further demonstrated that depletion of the transcription factor dFoxO or its partner Snail could inhibit APP‐ or AICD‐induced autophagy and axon degeneration. Finally, knockdown of *Atg1* or treatment with the autophagy inhibitor chloroquine effectively ameliorated APP‐ or AICD‐induced axon degeneration. In line with our results, previous studies have found that aberrant autophagy is associated with neurodegeneration (Nixon 2007; Zare‐Shahabadi et al. 2015). Furthermore, the study indicates that autophagy is required for Aβ secretion, and blocking autophagy by conditional knockout of Atg7 reduces Aβ plaque load in AD mice (Nilsson et al. 2013). Similarly, high brain cholesterol levels have been shown to promote autophagy‐dependent Aβ secretion and amyloid deposition in mice (Barbero‐Camps et al. 2018). Finally, the loss of FOXO3a in neural progenitor cells (NPCs) rescues AICD‐induced neurogenesis defects, including reduced NPC proliferation and differentiation, as well as depression‐like behavior in mice (Jiang et al. 2020). These findings provide valuable insights into the role of AICD in Alzheimer's disease pathology and suggest novel directions for further mechanistic and therapeutic exploration.

Consistent with our results that dFoxO is required for APP/AICD‐induced autophagy and axon degeneration, a previous study found that dFoxO/FOXO3 could physically interact with AICD and modulate APP/AICD‐triggered cell death (Wang et al. 2014). Our discovery that APP‐induced axon degeneration is dependent on AICD rather than Aβ appears to contradict previously published findings indicating that Aβ can be transported within neurites, contributing to axonal deficits in APP/PS1 (presenilin‐1) double‐transgenic mice (Christensen et al. 2014; Perneczky et al. 2014). We formulated a hypothesis that co‐expression of PS1, which releases both AICD and Aβ, might account for the discrepancy. In line with this hypothesis, Stokin and co‐workers have demonstrated that although the ratio of Aβ deposition remained unchanged, the level of axonal defects was suppressed when mutant PS1 was expressed in conjunction with APP (Stokin et al. 2008). In conclusion, our study not only uncovers a novel physiological function of AICD in APP‐induced axon degeneration, but also reveals the dFoxO/Snail‐Atg1 axis as an essential mediator of axon degeneration induced by APP or AICD.

## 4 Materials and Methods

### 4.1 Fly Strains

All fly stocks and crosses were maintained on standard *Drosophila* media at 25°C. The following strains were used for this study: *w* ^1118^, *UAS*‐Dcr2, *UAS‐BACE‐IR*, *UAS*‐*Psn‐IR*, *UAS*‐P35, *UAS*‐DIAP1, *UAS‐Atg7‐IR*, *UAS‐Atg12‐IR*, *dpr*‐Gal4, *APPL*‐Gal4 and UAS‐mCD8‐mCherry were previously described (Wang et al. 2014; Zhuang et al. 2020). *UAS*‐APP (hAPP695, 6700), *UAS*‐APP^ΔNPTY^ (29869), *UAS*‐APP^ΔAICD^ (29875), *UAS*‐GFP‐LAMP (42714), *UAS*‐mCD4‐GFP (35836), *UAS*‐EB1‐GFP (35512), *UAS*‐Atg1 (51655), *UAS‐Atg1‐IR* (26731) were obtained from the Bloomington stock center.

The *UAS*‐AICD fly strains were generated by PCR amplification (using KOD plus, TOYOBO) of a fragment from hAPP695 containing the first 17 amino acids of the signal peptide (SP) and the final 71 amino acids, which include the transmembrane (TM) region and the intracellular domain (ICD) (Figure S2A). The amplified fragment was cloned into the pUAST‐Myc vector. The primers used are as follows:

SP‐TM‐AICD‐FP (covering the SP and part of the TM):

5′TCCCCGCGGATGCTGCCCGGTTTGGCACTGCTCCTGCTGGCCGCCTGGACGGCTCGGGCGGGTGCAATCATTGGACTCATGGTGG‐3′

AICD‐RP: 5′‐TGCTCTAGAGTTCTGCATCTGCTCAAAGAACTTGTAGG‐3′

The plasmid was injected into *w* ^*1118*^ embryos, and transformants were selected based on eye color.

### 4.2 Axon Degeneration Scoreing System

In this study, the integrity and degeneration of axons were visualized using the fluorescent marker mCD8‐mCherry and quantitatively assessed. A defined region of the *Drosophila* wing nerve bundle (boxed in Figure S1) was analyzed using a standardized scoring system.

Score 0: Intact distal axons displayed continuous, smooth, and fibrous mCD8‐mCherry fluorescence, indicating no signs of axon degeneration.

Score 1: Following APP or AICD overexpression, axons exhibit initial signs of degeneration, including beading and minor fragmentation of the fluorescent signal.

Score 2: The mCD8‐mCherry fluorescent signals appeared fragmented, often accompanied by plaque‐like aggregation along the axons.

Score 3: The axonal structure was completely disrupted, with a near‐total loss of intact axons. This stage was characterized by a significant reduction in mCD8‐mCherry fluorescence intensity and signal presence.

### 4.3 Imaging of Fly Wings

To ensure consistency and minimize variability, we used only female flies in our experiments. Males exhibit greater phenotypic divergence, and the *APPL*‐Gal4 driver (X‐linked) combined with *UAS*‐APP causes male lethality. To maintain experimental uniformity, female flies were collected and reared separately until the indicated ages. For imaging, flies were anesthetized by CO_2_, and wings were carefully detached from the body on a CO_2_ anesthetizing pad. To ensure high‐quality images, wings were briefly washed in Wing Wash Buffer (WWB) containing 0.2% (vol/vol) Triton X‐100 and 4% (wt/vol) formaldehyde in 1× PBS to remove hydrophobicity, then placed on slides with alcohol/glycerol (1:3) buffer. All images were captured using an OLYMPUS BX51 microscope with consistent settings for all genotypes.

### 4.4 CQ Treatment

To prepare the CQ‐containing medium, 2.5 g of chloroquine (CQ) was dissolved in 25 mL of water, and then mixed with 475 mL of fruit fly medium to achieve a final concentration of 5 mg/mL CQ. A similar method was used to prepare medium containing 2 mg/mL CQ. Genotyped *Drosophila* collected after hybridization was reared on CQ‐supplemented food and transferred to fresh CQ‐containing medium every 2–3 days.

### 4.5 LysoTracker Red Staining

Lysosomal activity, serving as a marker of autophagy in *Drosophila* peripheral nerves, was detected by the LysoTracker Red Kit (Beyotime, C1046). Peripheral nerves were dissected from third‐instar larvae in PBS, and incubated with LysoTracker Red (1:3000) for 15 min at 37°C. Samples were then washed three times with PBS prior to imaging.

### 4.6 RT‐qPCR

Total RNA was extracted using Eastep Super (Shanghai Promega), and RT‐qPCR was performed using the SYBR Green PCR Premix Kit (TaKaRa). Primers used were as follows:

rp49‐F: 5′‐CCACCAGTCGGATCGATATGC‐3′

rp49‐R: 5′‐CTCTTGAGAACGCAGGCGACC‐3′

dFoxO‐F: 5′‐CGAGTTGGACAGTACAAAGGC‐3′

dFoxO‐R: 5′‐TGCATTCGCATTCTGTATAGCC‐3′

Atg1‐F: 5′‐CGTCAGCCTGGTCATGGAGTA‐3′

Atg1‐R: 5′‐TAACGGTATCCTCGCTGAG‐3′

### 4.7 Immunostaining

Adult fly brains were dissected in PBS solution and fixed with 4% formaldehyde for 40 min, followed by three washes in PBT (PBS with 0.3% Tween‐20). The dissected brains were blocked with 5% normal donkey serum in PBT, and incubated with primary antibodies at 4°C overnight. After washing, brains were incubated with secondary antibodies at room temperature for 2 h. The primary antibody was mouse anti‐myc (Cell Signaling Technology, 2276, 1:200); the secondary antibody was goat anti‐mouse Cy3 (Life technologies, A11032, 1:1000).

### 4.8 Western Blotting

Adult fly heads or wings were collected and homogenized in lysis buffer using a motorized pestle. The lysates were then centrifuged at 15,000 rpm for 10 min at 4°C. Proteins were separated by SDS‐PAGE following standard procedures. The primary antibodies were rabbit anti‐CD8 (abcam, ab217344, 1:2000), mouse anti‐myc (Cell Signaling Technology, 2276, 1:2000), rabbit anti‐α‐tubulin (Cell Signaling Technology, 2125, 1:2000) and rabbit anti‐GAPDH (Novus Biologicals, NB100‐56875, 1:2000). The secondary antibodies were goat anti‐rabbit‐HRP (Abways, AB0101, 1:2000) and goat anti‐mouse‐HRP (Abways, AB0102, 1:2000).

## Author Contributions

J.L., Y.Q., Y.P., C.L. and L.X. conceived and designed the experiments. J.L., Y.Q., Y.P., R.X., Y.S., L.Z., E.X. and C.L., performed the experiments, J.L., Y.Q., Y.P., R.X., Y.S., W.L., Q.Z., Z.L., C.L. and L.X. analyzed the data, L.X. supervised the study, J.L., Y.Q., Y.P., C.L. and L.X. wrote the manuscript. All authors approved the final manuscript.

## Funding

This work is supported by the National Natural Science Foundation of China (31970536, 32370891, 82303431), Shanghai Committee of Science and Technology (09DZ2260100) and China Postdoctoral Science Foundation (2023M730688).

## Conflicts of Interest

The authors declare no conflicts of interest.

## Supporting information


**Data S1:** acel70301‐sup‐0001‐Supinfo.docx.

## Data Availability

The data that support the findings of this study are available from the corresponding author upon reasonable request.
