# Supplementary material for: APP Induces AICD‐Mediated Autophagy‐Dependent Axon Degeneration
Source: Aging Cell. 2025 Nov 23;25(1):e70301. doi: 10.1111/acel.70301 (PMC12740101; doi:10.1111/acel.70301)
Supplement: Supplementary file 1 — Data S1: acel70301‐sup‐0001‐Supinfo.docx. [file ACEL-25-e70301-s001.docx]

**APP induces AICD-mediated autophagy-dependent axon degeneration**

Jingjing Luo^1,#^, Yu Qiu^1,#^, Yu Pan^1,#^, Ruihong Xu^1^, Yi Sun^1^, Yihao Sun^1^, Luming Zhuang^1^, Elleen Xue^2^, Wenzhe Li^1^, Qian Zhou^3^, Zhongwei Lv^1^, Chenglin Li^1,*^and Lei Xue^1,*^

^1^ Department of Nuclear Medicine, Shanghai 10th People’s Hospital, Shanghai Key Laboratory of Signaling and Diseases Research, School of Life Science and Technology, Tongji University, Shanghai, China

^2^ Mathey College, Princeton University, Princeton, New Jersey, USA

^3^ The First Rehabilitation Hospital of Shanghai, School of Medicine, Tongji University, Shanghai, China

^#^ These authors contributed equally to this work

^*^Corresponding author:

Lei Xue, E-mail: [lei.xue@tongji.edu.cn](mailto:lei.xue@tongji.edu.cn)

Chenglin Li, E-mail: [lichenglin@tongji.edu.cn](mailto:lichenglin@tongji.edu.cn)

**Supplementary Information**

Supplementary Figures

Detailed Genotypes

**Luo et al., Figure S1**

**
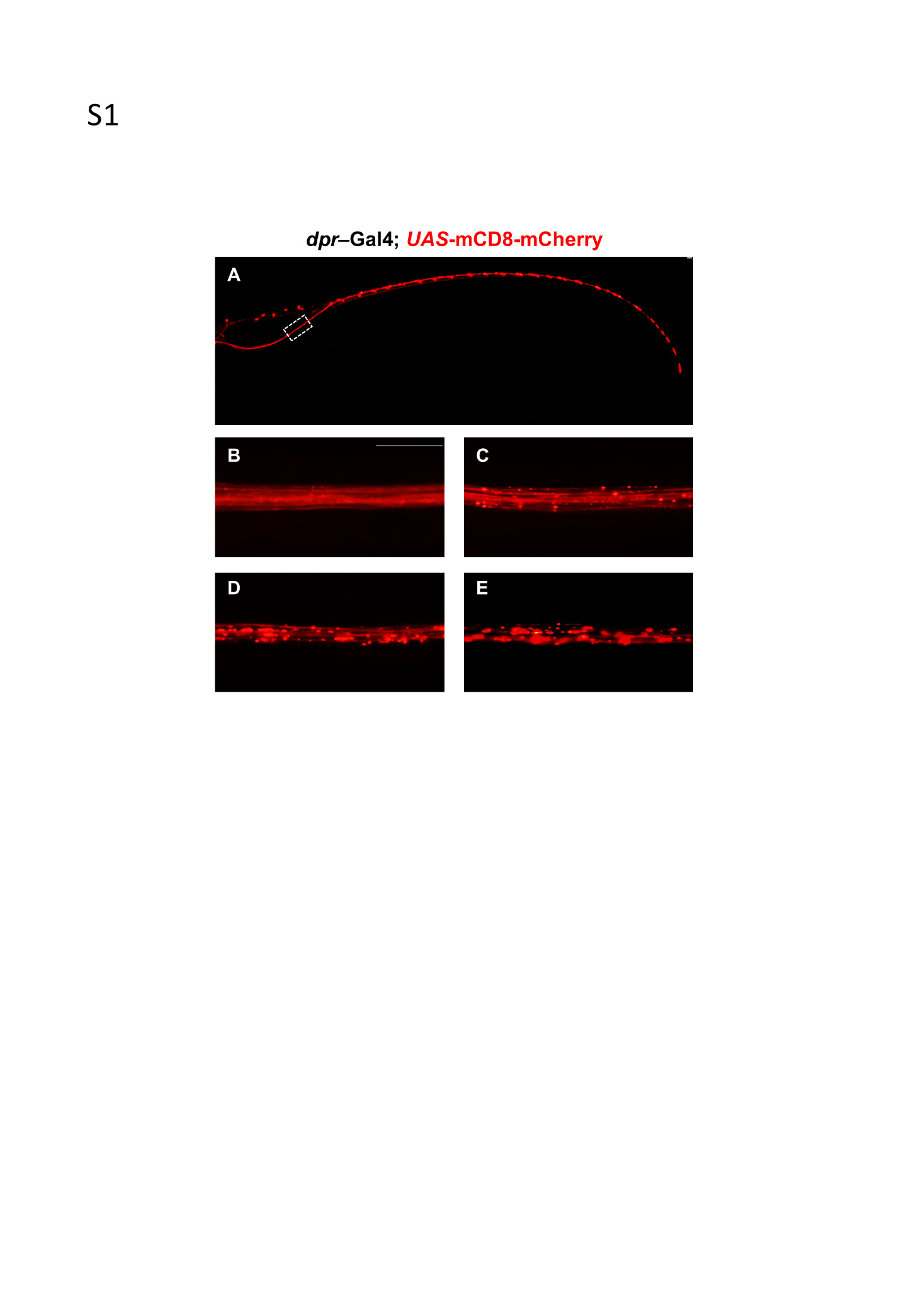
**

**Figure S1. The *Drosophila* wing nerve and axon degeneration evaluation system**

(A) The *Drosophila* wing nerve, highlighted by mCherry expression driven by *dpr*-Gal4 driver, with the wing arch outlined in white box. Scale bar: 200μm. (B-E) Examples of axon degeneration, visualized by mCherry, in the wing arch showing different categories of axon degeneration. Score 0: smooth and continuous axons (B); Score 1: axons with beading and minor fragmentation (C); Score 2: discontinuous axons with aggregated plaques (D); Score 3: axon structure completely destroyed, with a significant loss of mCD8-mCherry fluorescent signal (E). Scale bar: 20μm.

**
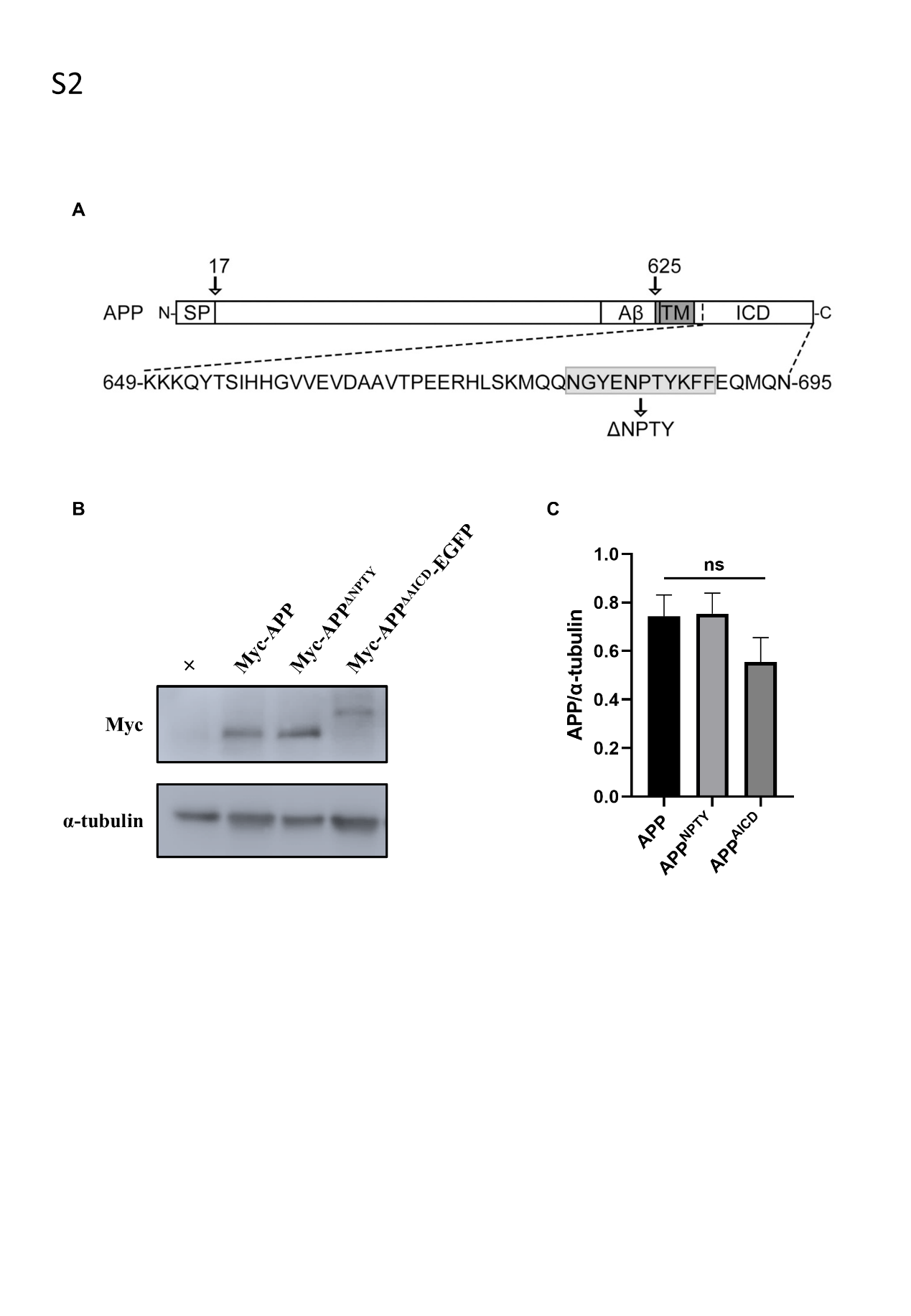
Luo et al., Figure S2**

**Figure S2. Expression of full length and truncated APP proteins**

(A) Schematic representation of full-length human APP695, highlighting the location of the NPTY motif. SP: signal peptide; TM: transmembrane domain. (B) Immunoblot analysis showing the expression levels of full-length and truncated APP proteins. (C) Statistical analysis of the APP/α-tubulin ratio from panel B. Each experiment was performed with more than three biologically independent replicates. Data are presented as mean ± SEM. ns represents no significant difference.

**
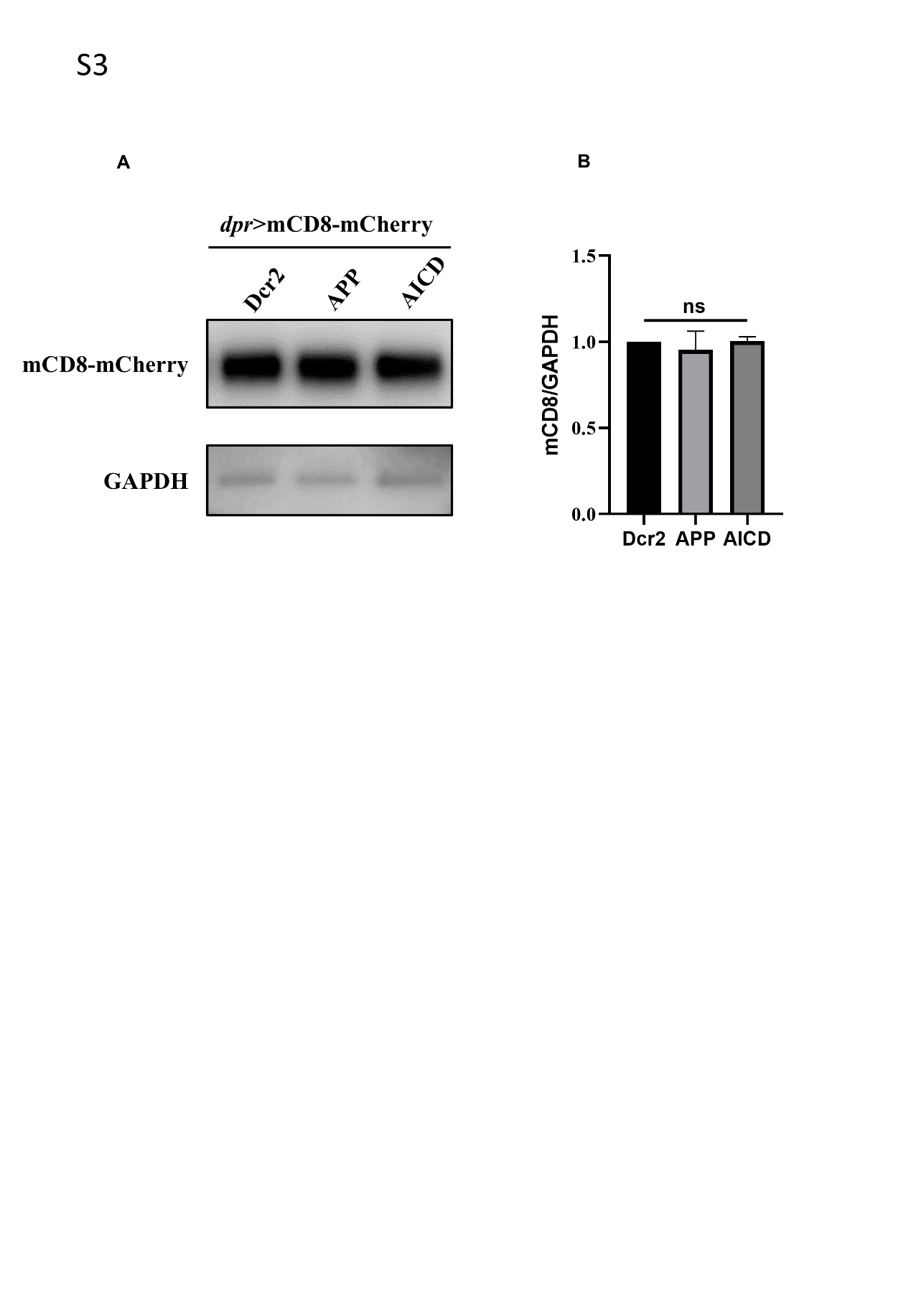
Luo et al., Figure S3**

**Figure S3. APP or AICD expression does not affect** **mCD8-mcherry protein levels**

(A) Immunoblot analysis of mCD8-mcherry protein levels in adult wing with an mCD8 antibody. Ectopic expression of APP or AICD, driven by *dpr*-Gal4, did not alter mCD8-mcherry protein levels. (B) Statistical analysis of the mCD8/GAPDH ratio from panel A. Data are presented as mean ± SEM. ns represents no significant difference.

**
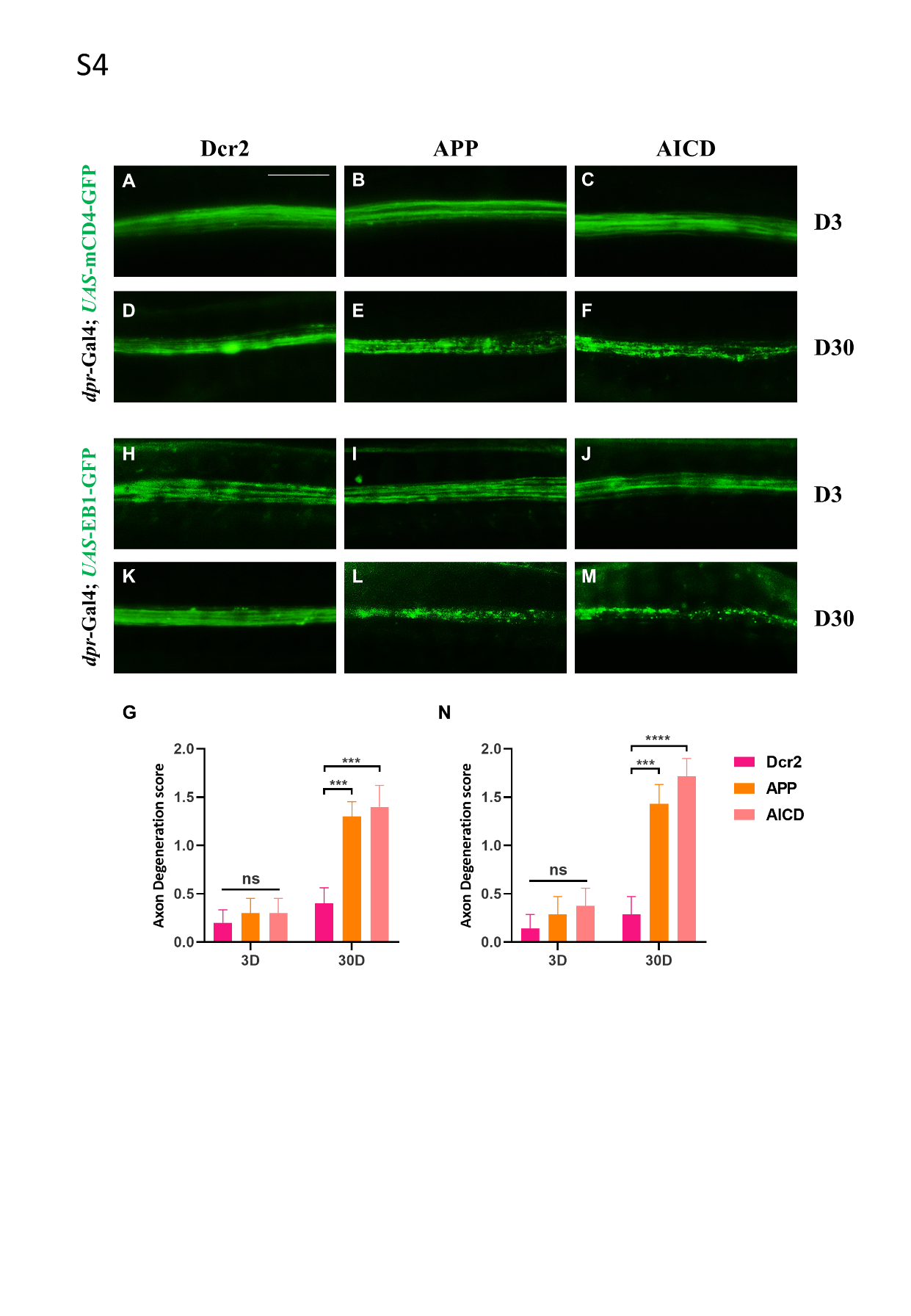
Luo et al., Figure S4**

**Figure S4. APP and AICD induce age-dependent axon degeneration**

(A-F) mCD4-GFP was expressed in the adult wing arch by *dpr*-Gal4. Compared with the control (A,D), APP or AICD overexpression showed normal axonal morphology at 3 days of age (B-C), while induced axon degeneration by day 30 (E-F). (G) Quantification of axon degeneration across different genotypes (n=10 per phenotype). (H-M) EB1-GFP was expressed in the adult wing arch via *dpr*-Gal4. No significant axon degeneration was observed in 3-day-old flies for any genotype (H-J). On day 30, compared with controls (K), expression of APP (L) or AICD (M) led to prominent axon degeneration. (N) Quantification of axon degeneration in different genotypes (n≥8 per phenotype). Data are presented as mean ± SEM. ns indicates no significant difference; *** p < 0.001; and **** P < 0.0001. Scale bar: 20 μm.

**
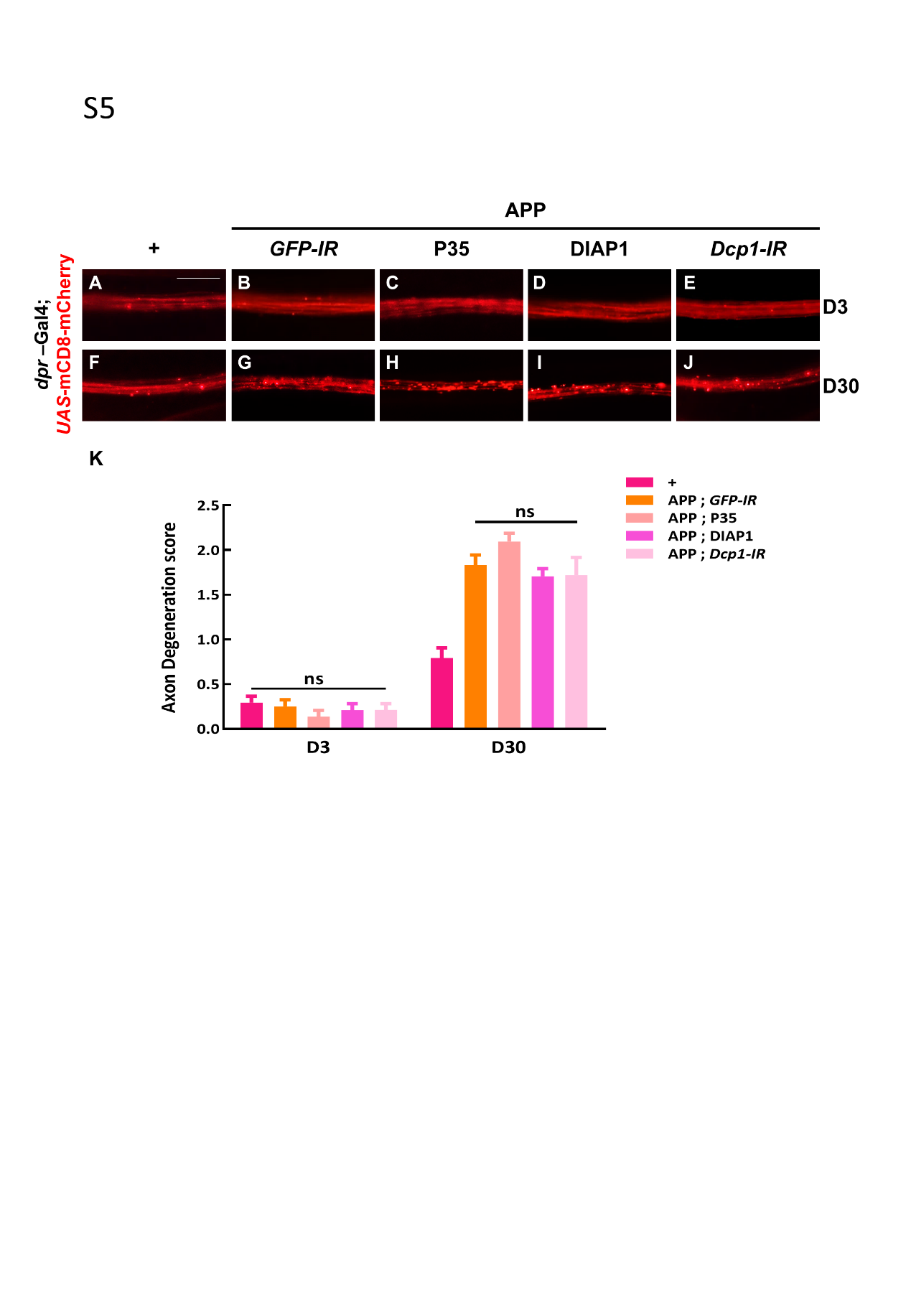
Luo et al., Figure S5**

**Figure S5. Inhibition of apoptosis does not suppress APP-induced axon degeneration**

(A-J) Visualization of the wing arch in adult females. On day 3, axons appeared smooth and continuous across all genotypes (A-E). On day 30, compared with the control group (G), overexpression of P35 (H) or DIAP1 (I), or knockdown of *Dcp1* (J), did not inhibit APP-induced axonal degeneration. (K) Statistical analysis of axon degeneration across different genotypes (n>15 per phenotype). Values are expressed as mean ± SEM, ns denotes no significant difference. Scale bar: 20 μm.

**
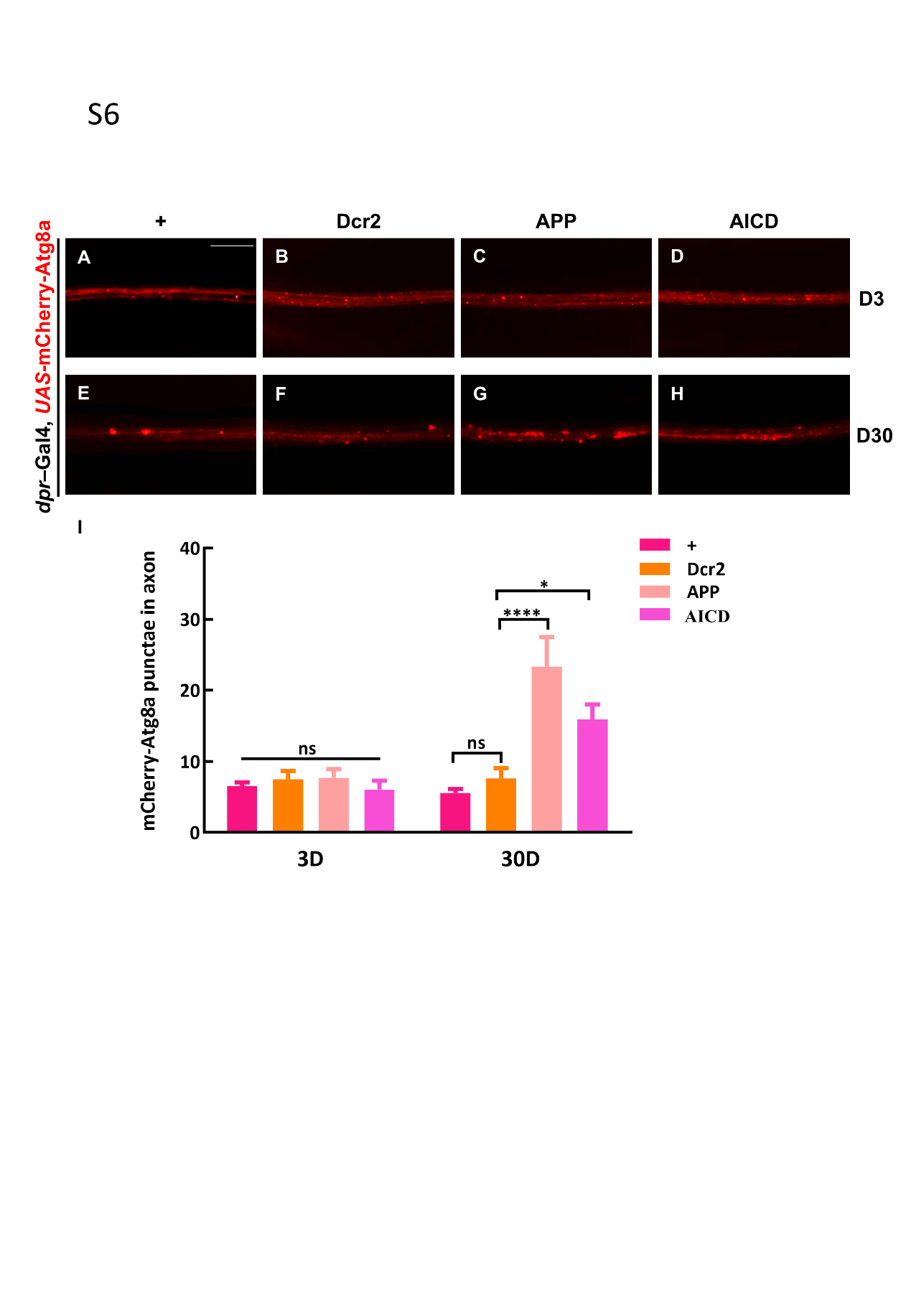
Luo et al., Figure S6**

**Figure S6. Overexpression of APP or AICD induces autophagosome accumulation in axons**

(A-H) Depiction of the wing arch in adult females. At day 3, a similar number of mCherry-Atg8a puncta was observed in both control groups (A, B) and flies expressing APP (C) or AICD (D) driven by *dpr*-Gal4. By day 30, compared with the controls (E, F), expression of APP (G) or AICD (H) led to an increased number of mCherry-Atg8a puncta. (I) Statistical analysis of mCherry-Atg8a puncta numbers across different genotypes (n>15 per phenotype). Values are expressed as mean ± SEM, ns denotes not significant; * represents P < 0.05, and **** represents P < 0.0001. Scale bar: 20 μm.

**
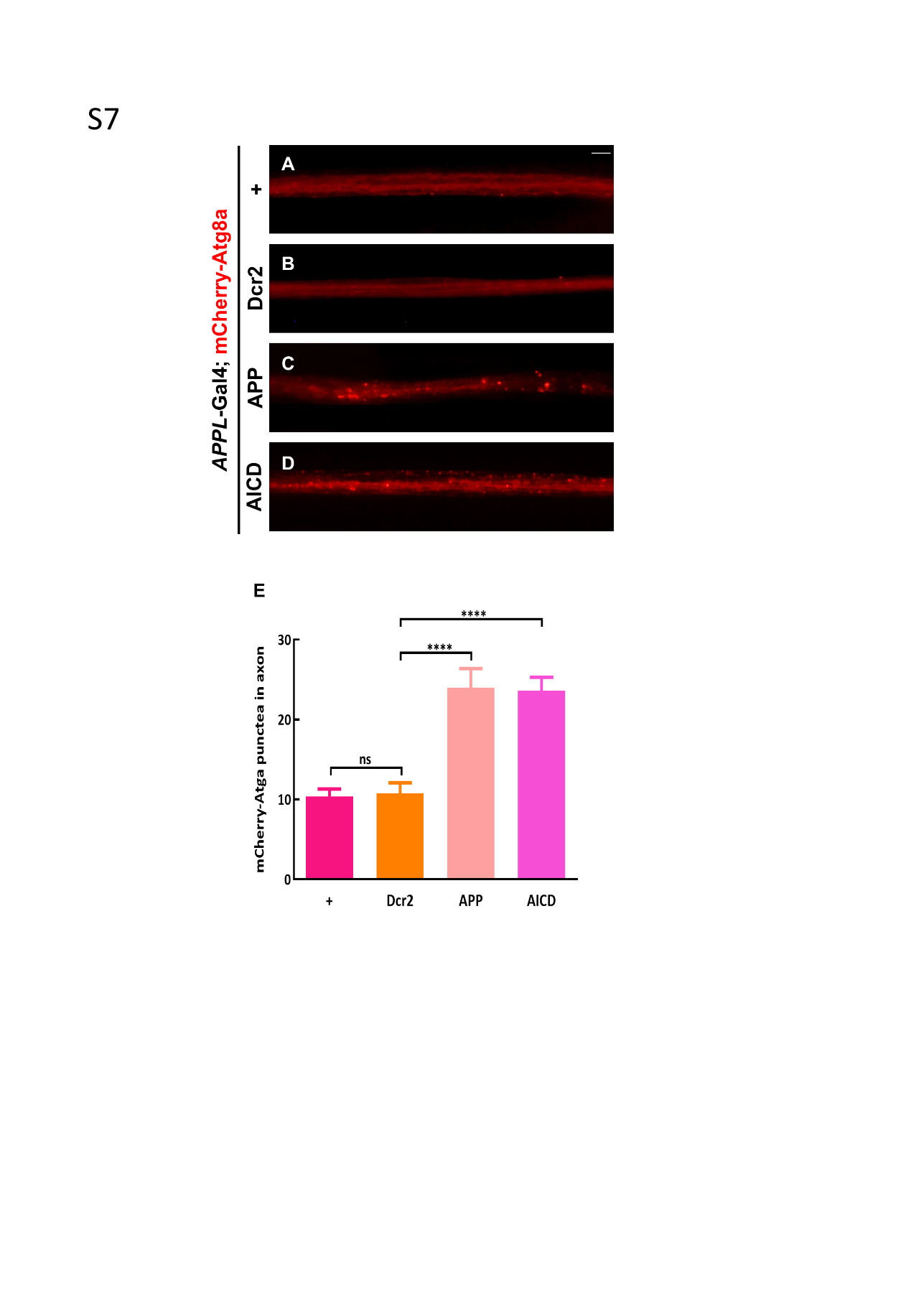
Luo et al., Figure S7**

**Figure S7. Overexpression of APP induces autophagosome accumulation in 3rd instar larval peripheral nerves**

(A-D) Peripheral nerves of 3rd instar larvae. Compared with control groups (A, B), an increased number of mCherry-Atg8a puncta was observed upon expression of APP (C) or AICD (D). (E) Statistical analysis of mCherry-Atg8a puncta numbers across different genotypes (n>15 per phenotype). Values are expressed as mean ± SEM, ns denotes not significant, **** represents P < 0.0001. Scale bar: 20 μm.

**
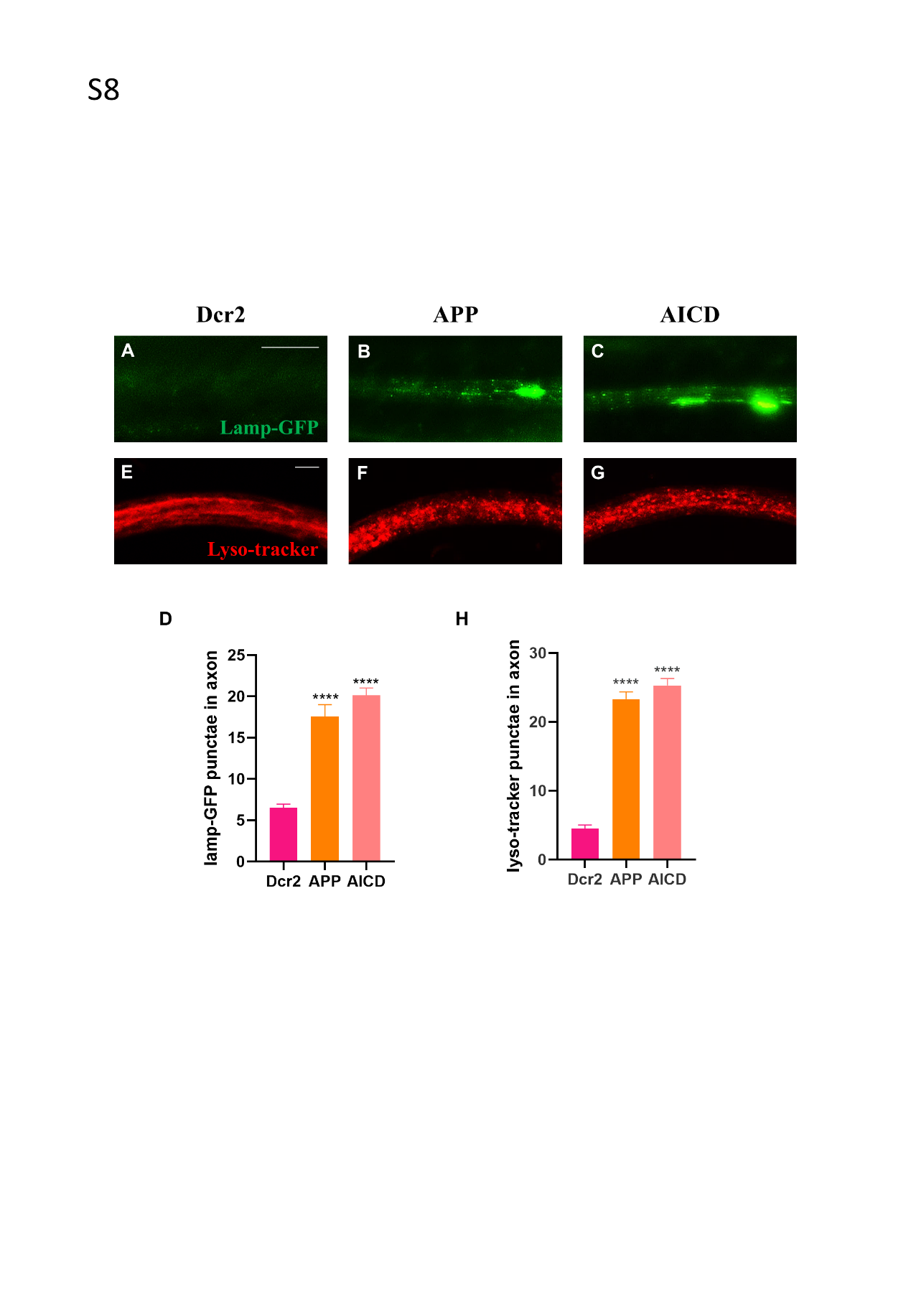
Luo et al., Figure S8**

**Figure S8. Overexpression of APP or AICD induce autolysosome accumulation**

(A-C) Visualization of the wing arch in adult flies. Comapred with the control (A), ectopic expression of APP (B) or AICD (C), driven by the *dpr*-Gal4 driver, led to an increased number of Lamp-GFP puncta at day 15. (D) Quantification of Lamp-GFP puncta across different genotypes (n≥7 per phenotype). (E-G) Peripheral nerves of 3rd instar larvae. Compared with the control groups (E), expression of APP (F) or AICD (G) resulted in increased Lyso-tracker puncta. (H) Statistical analysis of Lyso-tracker puncta across different genotypes (n=10 per phenotype).Values are expressed as mean ± SEM, **** represents P < 0.0001. Scale bar: 20 μm.

**
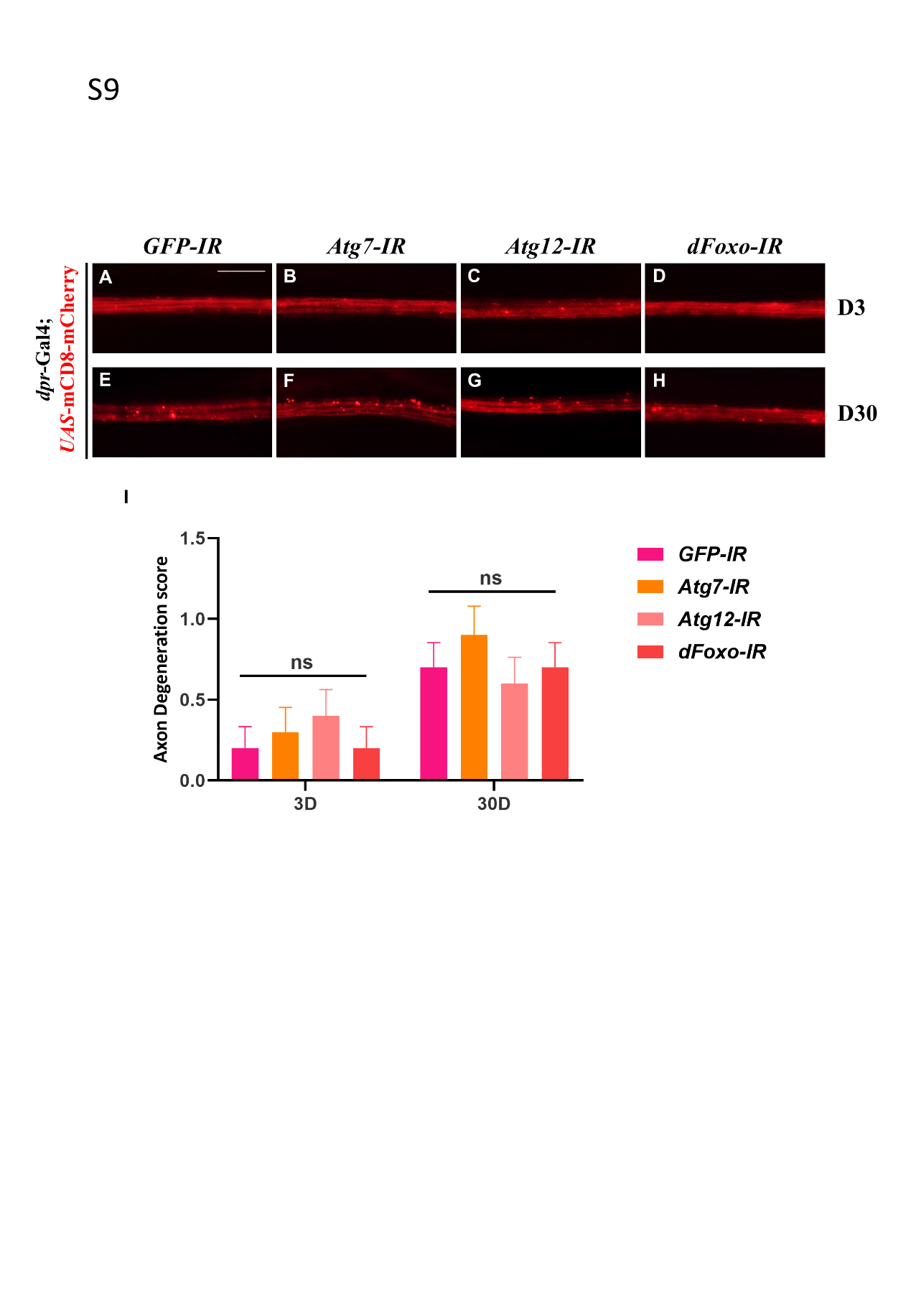
Luo et al., Figure S9**

**Figure S9. Expression of *Atg7-IR*, *Atg12-IR*, or *dFoxO-IR* alone does not induce axon degeneration**

(A-H) Visualization of the wing arch in adult flies. Compared with the controls (A, E), no significant axon degeneration was observed in either 3-day-old or 30-day-old flies expressing *Atg7-IR* (B, F), *Atg12-IR* (C, G), or *dFoxO-IR* (D, H) driven by *dpr*-Gal4. (I) Statistical analysis of axon degeneration for all genotypes (n=10 per phenotype). Values are shown as mean ± SEM, ns represents not significant. Scale bar: 20 μm.

**
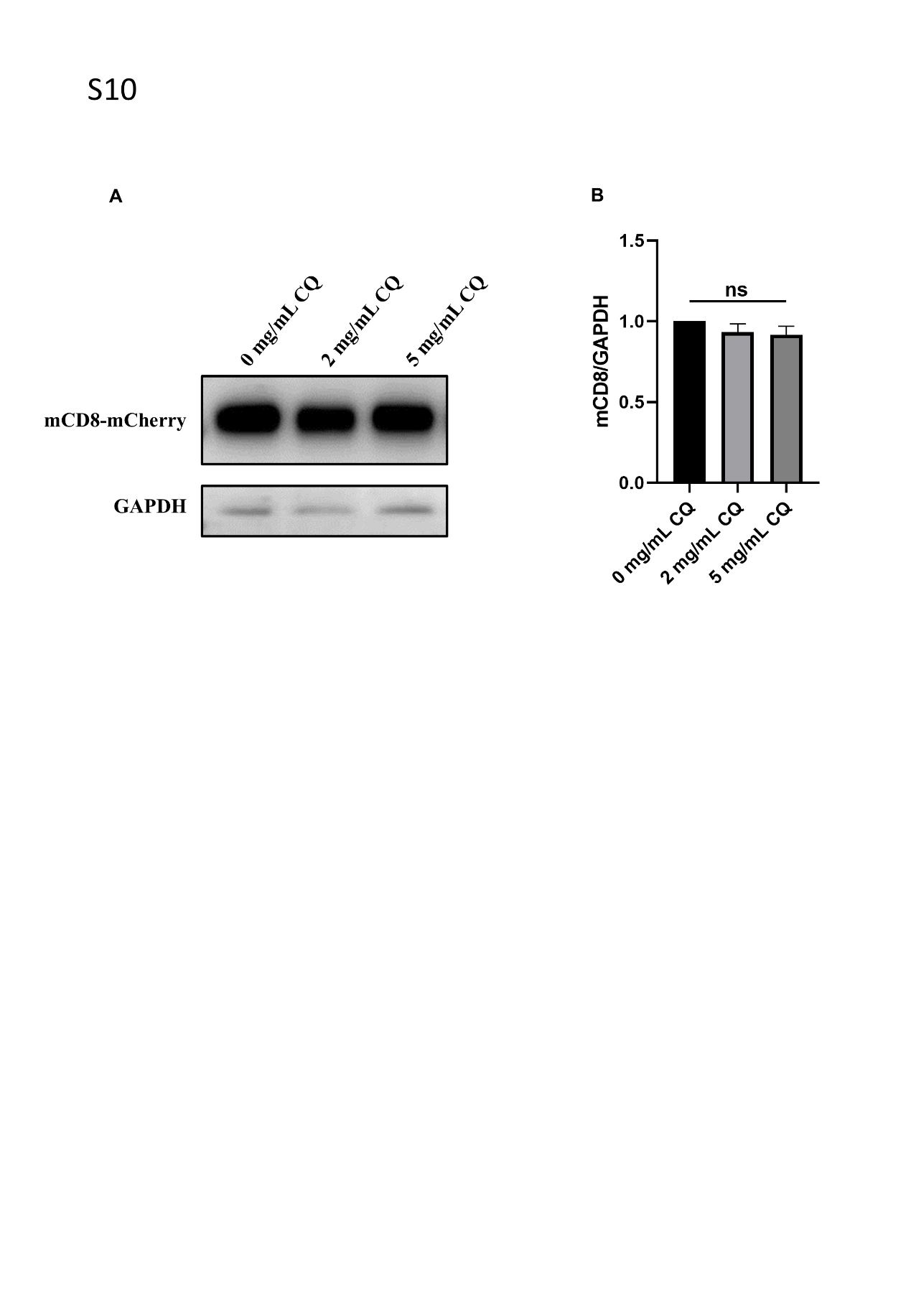
Luo et al., Figure S10**

**Figure S10.** **Chloroquine does not affect mCD8-mCherry protein level**

(A) Immunoblot analysis of mCD8-mcherry protein level in adult wing using an mCD8 antibody. Treatment with different concentration of Chloroquine did not alter mCD8-mCherry protein level. (B) Statistical analysis of the mCD8/GAPDH ratio from panel A. Values are presented as mean ± SEM. ns represents not significant.

**
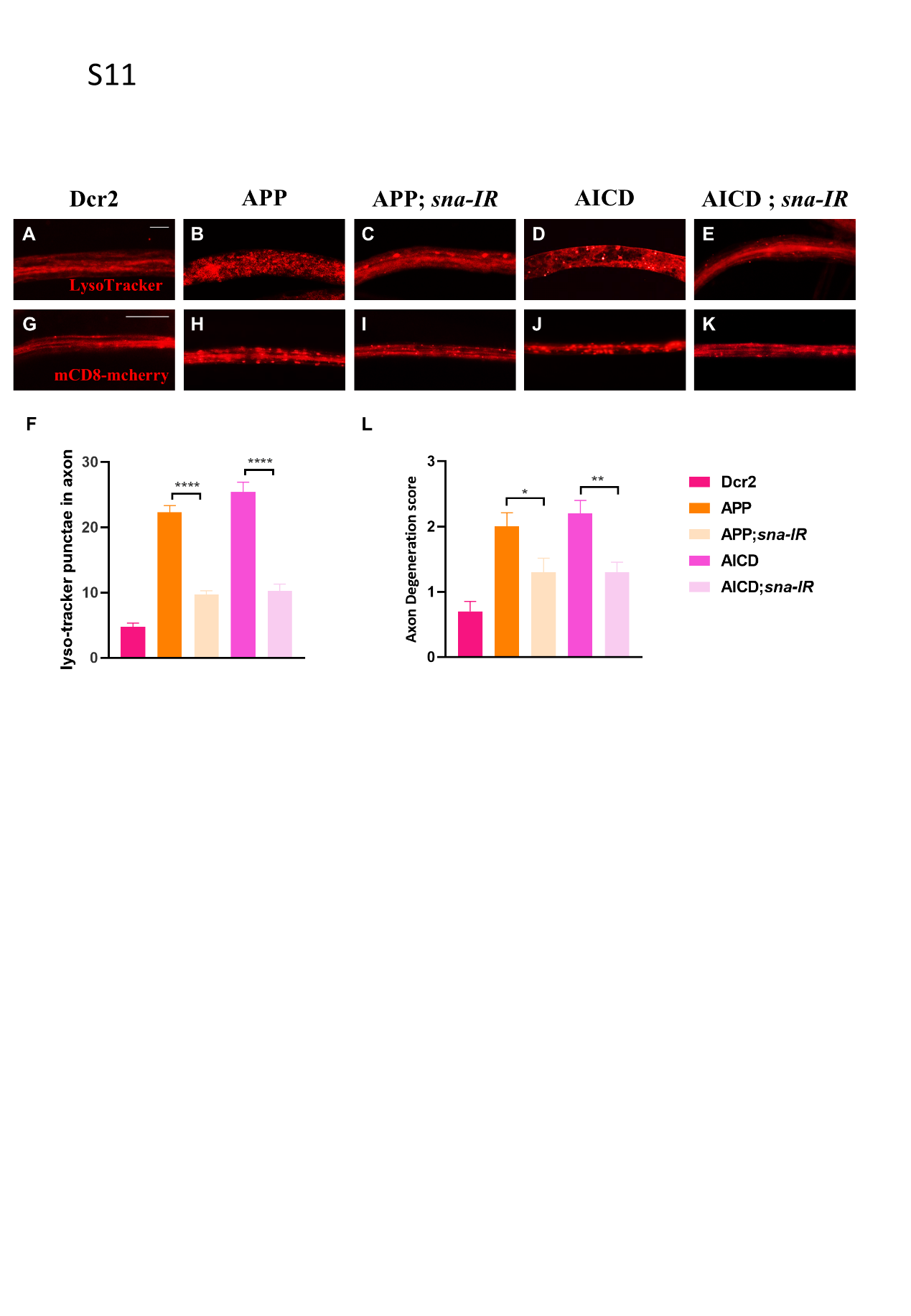
Luo et al., Figure S11**

**Figure S11. Snail contributes to APP- or AICD-induced autophagy-dependent axon degeneration**

(A-E) Peripheral nerves of 3rd instar larvae. Compared with the control (A), overexpression of APP (B) or AICD (D), driven by *APPL*-Gal4, activated autophagy, as indicated by increased LysoTracker puncta. This effect was suppressed by knockdown of *sna* (C, E). (F) Statistical analysis of LysoTracker puncta numbers across different genotypes (n=10 per phenotype). (G-K) Wing arch of 15-day-old adult flies. Compared with the controls (G), ectopic APP- or AICD-induced axonal degeneration (H, J) was significantly suppressed by knockdown of *sna* (I, K). (L) Quantification of axonal degeneration across different genotypes. Values are expressed as mean ± SEM, ns denotes not significant, * represents P < 0.05, **p < 0.01 and **** represents P < 0.0001. Scale bar: 20 μm.

**
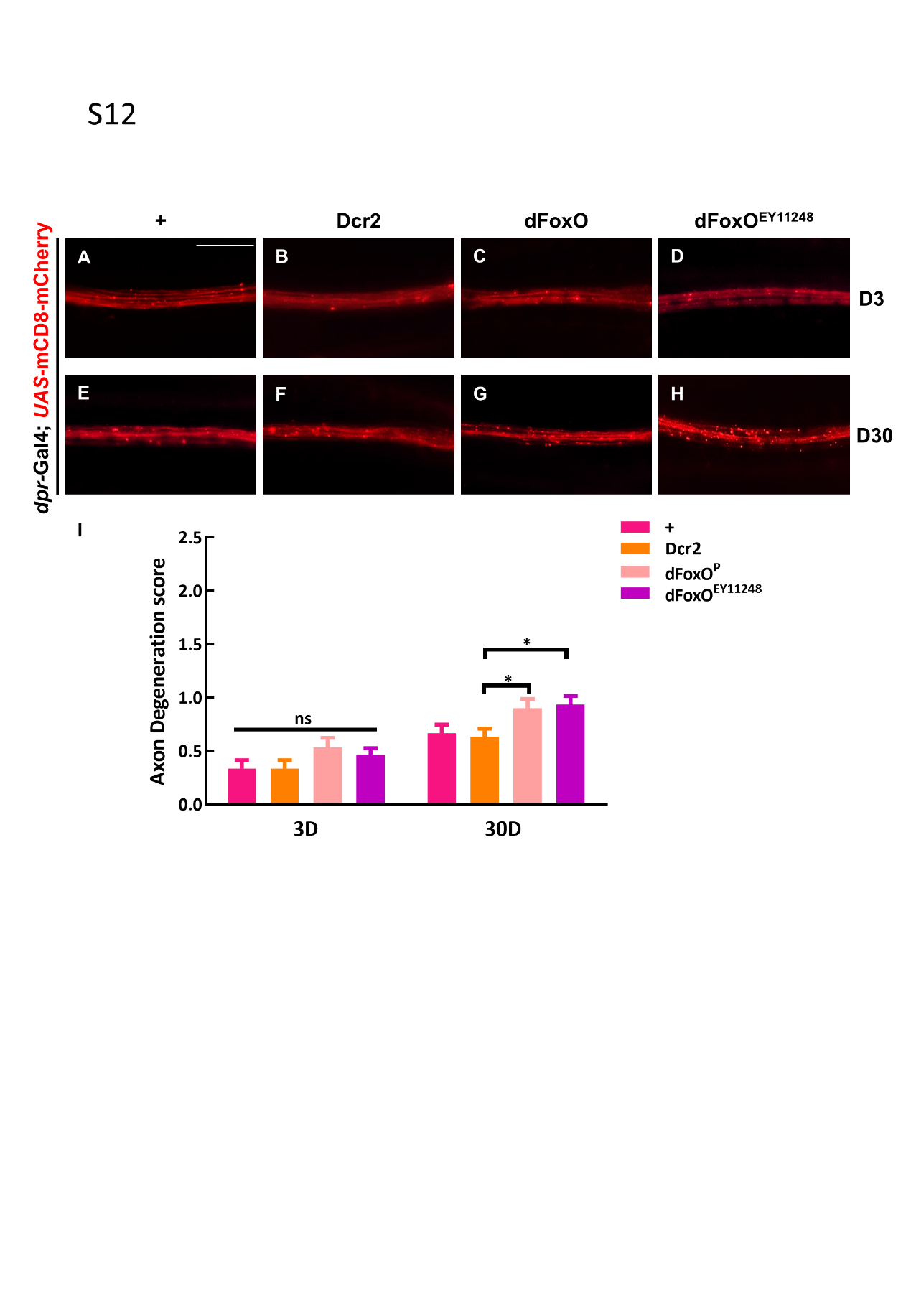
Luo et al., Figure S12**

**Figure S12. Overexpression of dFoxO is sufficient to trigger axon degeneration**

(A-D) Depiction of the wing arch in adult females. The axons of different genotype were smooth and continuous on day 3 (A-D). Ectopic expression of dFoxO induced axon degeneration (G, H) when compared with the control (E, F) on day 30. (I) Statistical analysis of axon degeneration of different genotypes (n>15 per phenotype). Values are expressed as mean ± SEM, ns represents not significant, * represents P < 0.05. Scale bar: 20 μm.

**
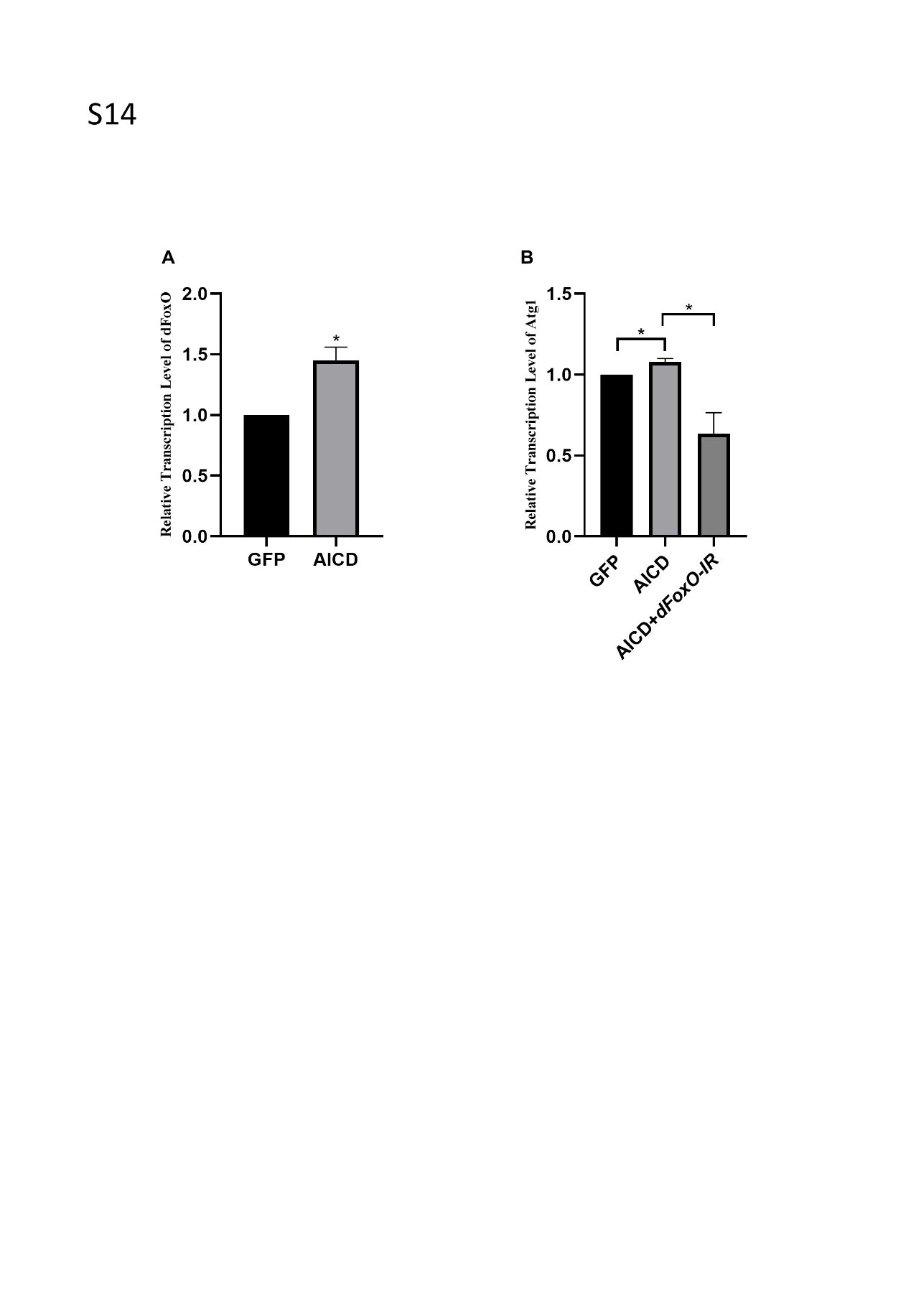
Luo et al., Figure S13**

**Figure S13. AICD Overexpression increases the transcription of *dFoxO* and *Atg1***

(A) RT-qPCR analysis showing that overexpression of AICD elevates *dFoxO* mRNA levels. (B) Overexpression of AICD also increased *Atg1* mRNA levels, an effect that is suppressed by *dFoxO* knockdown, as measured by RT-qPCR. Values are expressed as mean ± SEM, ns represents not significant, * represents P < 0.05.

**
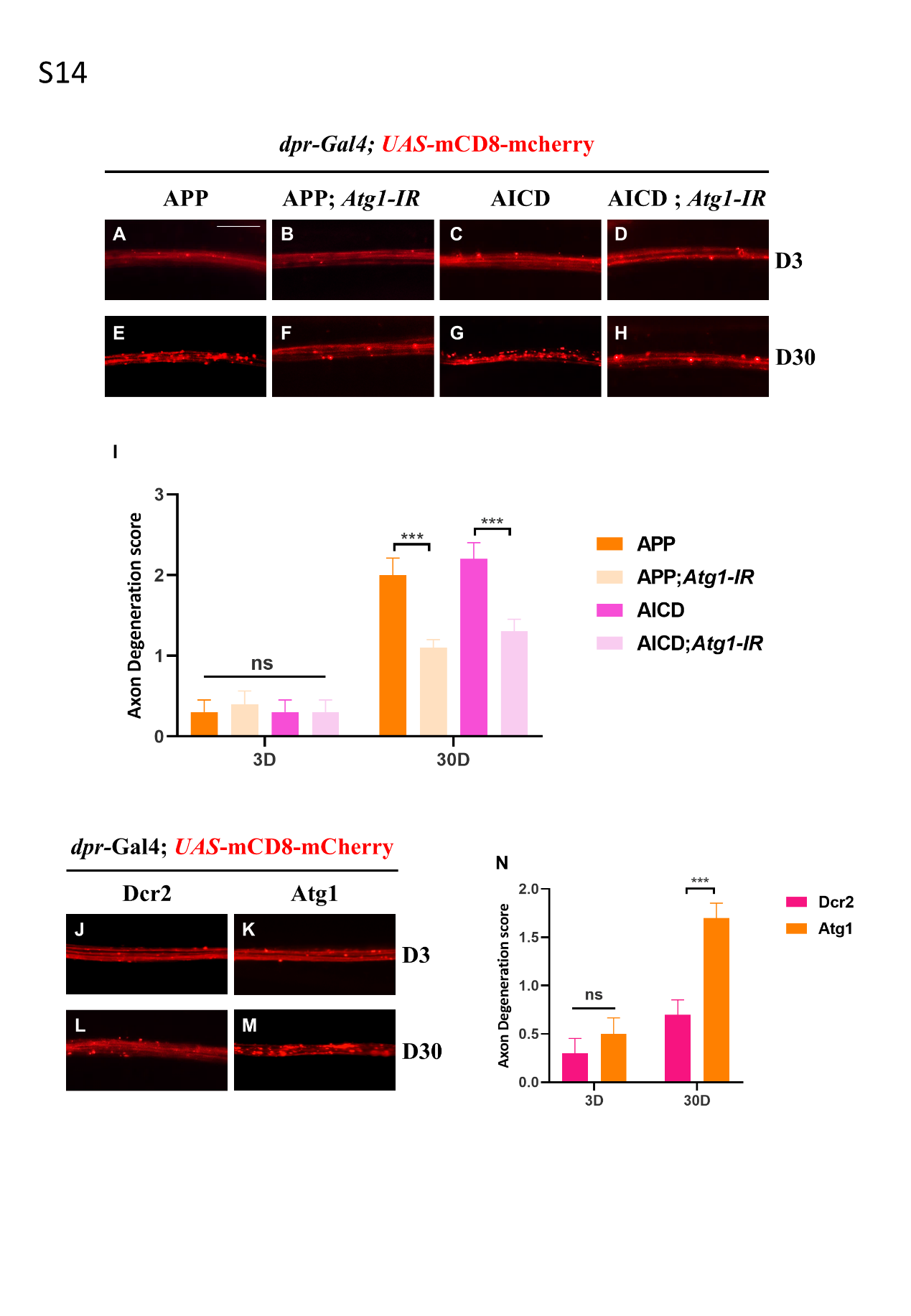
Luo et al., Figure S14**

**Figure S14. Atg1 is necessary and sufficient for APP-induced axon degeneration**

(A-H) Depiction of the wing arch in adult flies. At day 3, axons appeared smooth and continuous across all genotypes (A–D). By day 30, APP- (E) or AICD-induced (G) axon degeneration was significantly suppressed by *Atg1* depletion (F, H). (I) Quantification of axon degeneration in different genotypes (n=10 per phenotype). (J-M) Wing arch of adult flies showing no significant difference between control (J) and Atg1-expressing flies at day 3 (K). However, by day 30, overexpression of Atg1 resulted in marked axon degeneration (M) compared with controls (L). (N) Quantification of axon degeneration in the indicated genotypes (n = 10 per group). Values are expressed as mean ± SEM, ns represents not significant, *** represents p < 0.001. Scale bar: 20 μm.

**
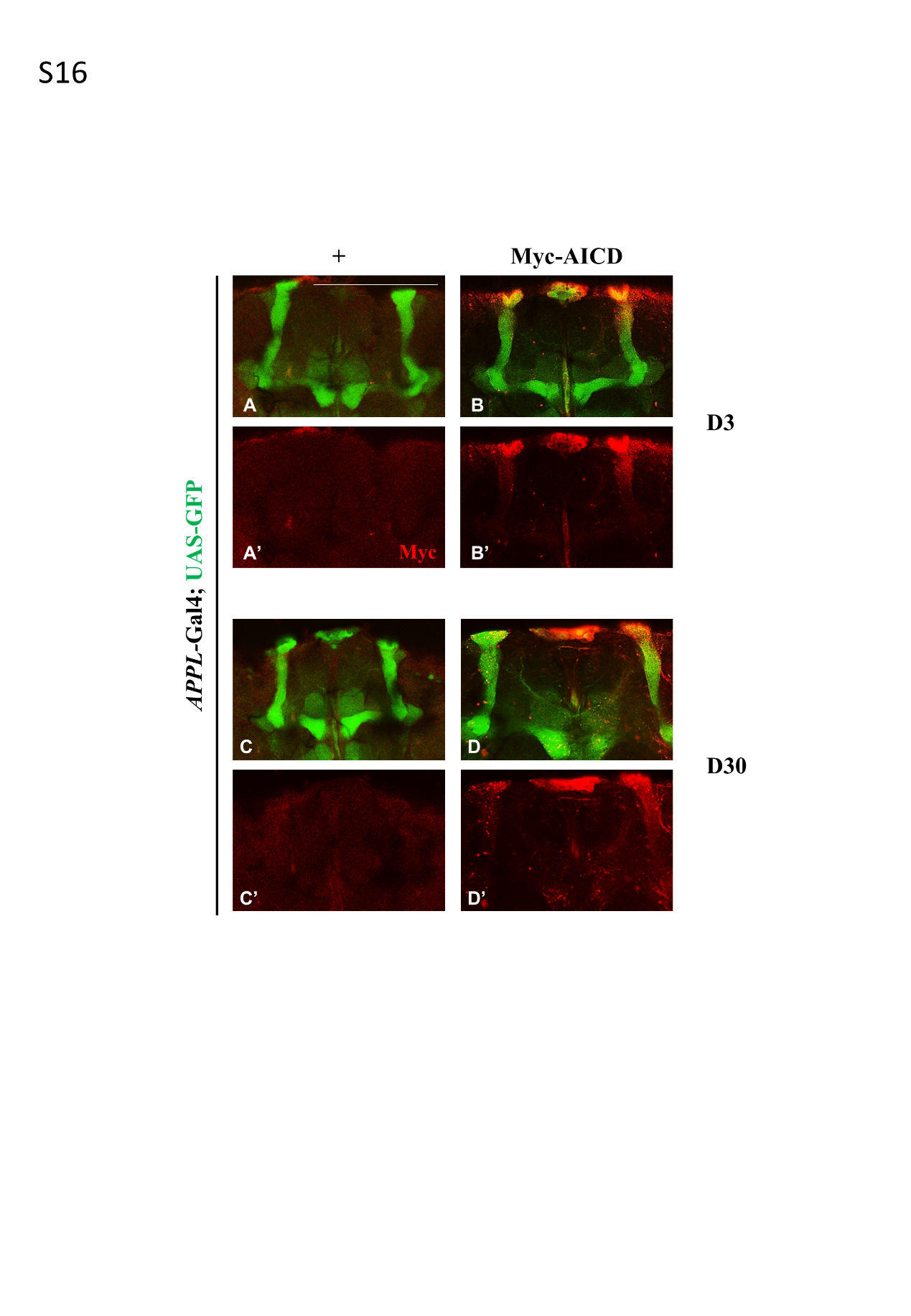
Luo et al., Figure S15**

**Figure S15. AICD localizes to axons and forms aggregates with age**

(A–D) Fluorescent micrographs of adult *Drosophila* brains showing the mushroom bodies (MB). Compared with controls (A, A’, C, C’), neuronally expressed AICD localized to the MB lobes formed by axons (B, B’, D, D’), as detected by an anti-Myc antibody. Notably, AICD formed visible aggregates within axons by day 30 (D, D’). Scale bar: 20 μm.

**Detailed Genotypes for All Figures**

**Figure 1**

(A, D, G) *dpr-Gal4/+; UAS-mCD8-mCherry/+*

(B, E, H) *dpr-Gal4/+; UAS-mCD8-mCherry/UAS-Dcr2*

(C, F, I) *dpr-Gal4/UAS-Myc-APP; UAS-mCD8-mCherry/+*

**Figure 2**

(A, D) *dpr-Gal4/UAS-Myc-APP; UAS-mCD8-mCherry/UAS-GFP-IR*

(B, E) *dpr-Gal4/UAS-Myc-APP; UAS-mCD8-mCherry/UAS-Bace-IR*

(C, F) *dpr-Gal4/UAS-Myc-APP; UAS-mCD8-mCherry/UAS-Psn-IR*

(G, J) *dpr-Gal4/UAS-Myc-APP; UAS-mCD8-mCherry/+*

(H, K) *dpr-Gal4/+; UAS-mCD8-mCherry/UAS-Myc-APP^ΔAICD^-EGFP*

(I, L) *dpr-Gal4/+; UAS-mCD8-mCherry/UAS-Myc-APP^ΔNPTY^*

**Figure 3**

(A, D) *dpr-Gal4/+; UAS-mCD8-mCherry/UAS-Dcr2*

(B, E) *dpr-Gal4/UAS-Myc-APP; UAS-mCD8-mCherry/+*

(C, F) *dpr-Gal4/UAS-Myc-AICD; UAS-mCD8-mCherry/+*

**Figure 4**

(A, D) *dpr-Gal4/UAS-Myc-APP; UAS-mCD8-mCherry/UAS-GFP-IR*

(B, E) *dpr-Gal4/UAS-Myc-APP; UAS-mCD8-mCherry/UAS-Atg7-IR*

(C, F) *dpr-Gal4/UAS-Myc-APP; UAS-mCD8-mCherry/UAS-Atg12-IR*

(G, J) *UAS-Myc-AICD/+;dpr-Gal4/+; UAS-mCD8-mCherry/UAS-GFP-IR*

(H, K) *UAS-Myc-AICD/+;dpr-Gal4/+; UAS-mCD8-mCherry/UAS-Atg7-IR*

(I, L) *UAS-Myc-AICD/+;dpr-Gal4/+; UAS-mCD8-mCherry/UAS-Atg12-IR*

**Figure 5**

(A, E, I, M, Q, U) *dpr-Gal4/+; UAS-mCD8-mCherry/+*

(B, F, J, N, R, V) *dpr-Gal4/+; UAS-mCD8-mCherry/UAS-Dcr2*

(C, G, K, O, S, W) *dpr-Gal4/UAS-Myc-APP; UAS-mCD8-mCherry/+*

(D, H, L, P, T, X) *UAS-Myc-AICD/+;dpr-Gal4/+; UAS-mCD8-mCherry/+*

**Figure 6**

(A, E) *dpr-Gal4/UAS-Myc-APP; UAS-mCD8-mCherry/UAS-GFP-IR*

(B, F) *dpr-Gal4/UAS-Myc-APP; UAS-mCD8-mCherry/UAS-dFoxO-IR*

(C, G) *dpr-Gal4/UAS-Myc-APP; UAS-mCD8-mCherry/dFoxO^Δ21^*

(D, H) *dpr-Gal4/UAS-Myc-APP; UAS-mCD8-mCherry/dFoxO^Δ94^*

**Figure 7**

(A, F) *dpr-Gal4,UAS-mCherry-Atg8a/+;* *UAS-Dcr2/+*

(B, G) *dpr-Gal4,UAS-mCherry-Atg8a/UAS-Myc-APP*

(C, H) *dpr-Gal4,UAS*-*mCherry-Atg8a/UAS-Myc-APP*; *UAS-dFoxO-IR*/+

(D, I) *dpr-Gal4,UAS*-*mCherry-Atg8a/UAS-Myc-AICD*

(E, J) *dpr-Gal4,UAS*-*mCherry-Atg8a/UAS-Myc-AICD; UAS-dFoxO-IR*/+

**Figure S1**

(A-E) *dpr-Gal4/+; UAS-mCD8-mCherry/+*

**Figure S2**

(B) From left to right: (1) *APPL*-*Gal4*/+ (2) *APPL*-*Gal4*/+; *UAS*-*Myc*-APP/+ (3) *APPL*-*Gal4*/+; *UAS*-*Myc-APP^ΔNPTY^*/+ (4) *APPL-Gal4*/+; *UAS*-*Myc-APP^ΔAICD^-EGFP*

**Figure S3**

(A) From left to right: (1) *dpr-Gal4,UAS-mCD8-mCherry*/+; *UAS*-*Dcr2*/+ (2) *dpr-Gal4,UAS-mCD8-mCherry*/*UAS*-*Myc-*APP (3) *dpr-Gal4,UAS-mCD8-mCherry*/*UAS*-*Myc-*AICD/+

**Figure S4**

(A, D) *dpr-Gal4*/+; *UAS*-*mCD4-GFP/UAS-Dcr2*

(B, E) *dpr-Gal4*/*UAS-Myc-APP*; *UAS*-*mCD4-GFP/*+

(C, F) *dpr-Gal4*/*UAS-Myc-AICD*; *UAS*-*mCD4-GFP/*+

(H, K) *dpr-Gal4*/+; *UAS*-*EB1-GFP/UAS-Dcr2*

(I, L) *dpr-Gal4*/*UAS-Myc-APP*; *UAS*-*EB1-GFP/*+

(J, M) *dpr-Gal4*/*UAS-Myc-AICD*; *UAS*-*EB1-GFP/*+

**Figure S5**

(A, F) *dpr*-*Gal4/+; UAS-mCD8-mCherry/+*

(B, G) *dpr-Gal4/UAS-Myc-APP; UAS-mCD8-mCherry/UAS-GFP-IR*

(C, H) *dpr-Gal4/UAS-Myc-APP; UAS-mCD8-mCherry/UAS-P35*

(D, I) *dpr-Gal4/UAS-Myc-APP; UAS-mCD8-mCherry/UAS-DIAP1*

(E, J) *dpr-Gal4/UAS-Myc-APP; UAS-mCD8-mCherry/UAS-Dcp1-IR*

**Figure S6**

(A, E) *dpr-Gal4, UAS-mCherry-Atg8a/+*

(B, F) *dpr-Gal4, UAS-mCherry-Atg8a/UAS-Dcr2*

(C, G) *dpr-Gal4, UAS-mCherry-Atg8a/UAS-Myc-APP*

(D, H) *UAS-Myc-AICD/+; dpr-Gal4, UAS-mCherry-Atg8a/+*

**Figure S7**

(A) *APPL-Gal4/+; UAS-mCherry-Atg8a/+*

(B) *APPL-Gal4/+; UAS-mCherry-Atg8a/UAS-Dcr2*

(C) *APPL-Gal4/+; UAS-mCherry-Atg8a/UAS-Myc-APP*

(D) *APPL-Gal4/UAS-Myc-AICD; UAS-mCherry-Atg8a/+*

**Figure S8**

(A) *dpr-Gal4,UAS-Lamp-GFP/+; UAS-Dcr2/+*

(B) *dpr-Gal4,UAS-Lamp-GFP/UAS-Myc-APP*

(C) *dpr-Gal4,UAS-Lamp-GFP/UAS-Myc-AICD*

(E) *APPL-Gal4/+; UAS-Dcr2/+*

(F) *APPL-Gal4/+; UAS*-*Myc-APP/+*

(G) *APPL-Gal4/+; UAS*-*Myc-AICD/+*

**Figure S9**

(A, E) *dpr-Gal4,UAS-mCD8-mCherry/+*; *UAS-GFP-IR*/+

(B, F) *dpr-Gal4,UAS-mCD8-mCherry/+*; *UAS-Atg7-IR*/+

(C, G) *dpr-Gal4,UAS-mCD8-mCherry/+*; *UAS-Atg12-IR*/+

(D, H) *dpr-Gal4,UAS-mCD8-mCherry/+*; *UAS-dFoxO-IR*/+

**Figure S10**

(A) *dpr-Gal4,UAS-mCD8-mCherry/+; UAS**-Dcr2/+*

**Figure S11**

(A) *APPL*-*Gal4/+; UAS**-Dcr2/+*

(B) *APPL*-*Gal4/+; UAS**-Myc-APP/+*

(C) *APPL-Gal4/+; UAS-Myc-APP/+*; *UAS-sna-IR*/+

(D) *APPL*-*Gal4/+; UAS-Myc-AICD/+*

(E) *APPL*-*Gal4/+; UAS-Myc-AICD/+*; *UAS-sna-IR*/+

(G) *dpr-Gal4,UAS-mCD8-mCherry/+; UAS-Dcr2/+*

(H) *dpr-Gal4,UAS-mCD8-mCherry/UAS-Myc-APP*

(I) *dpr-Gal4,UAS-mCD8-mCherry/UAS-Myc-APP*; *UAS-sna-IR*/+

(J) *dpr-Gal4,UAS-mCD8-mCherry/UAS-Myc-AICD*

(K) *dpr*-*Gal4,UAS-mCD8-mCherry/UAS-Myc-AICD*; *UAS-sna-IR*/+

**Figure S12**

(A, E) *dpr-Gal4/+; UAS-mCD8-mCherry/+*

(B, F) *dpr-Gal4/+; UAS-mCD8-mCherry/UAS-Dcr2*

(C, G) *dpr-Gal4/+; UAS-mCD8-mCherry/UAS-dFoxO*

(D, H) *dpr-Gal4/+; UAS-mCD8-mCherry/dFoxO^EY11248^*

**Figure S14**

(A, E) *dpr-Gal4,UAS-mCD8-mCherry/UAS-Myc-APP*

(B, F) *dpr-Gal4,UAS-mCD8-mCherry/UAS-Myc-APP*; *UAS-**Atg1-IR*/+

(C, G) *dpr-Gal4,UAS-mCD8-mCherry/UAS-Myc-AICD*

(D, H) *dpr-Gal4,UAS-mCD8-mCherry/UAS-Myc-AICD*; *UAS-Atg1-IR*/+

(J, L) *dpr-Gal4,UAS-mCD8-mCherry/+; UAS-Dcr2/+*

(K, M) *dpr-Gal4,UAS-mCD8-mCherry/+; UAS-Atg1/+*

**Figure S15**

(A, A’, C, C’) *APPL*-*Gal4/+; UAS-GFP/+*

(B, B’, D, D’) *APPL*-*Gal4/+; UAS-Myc-AICD/+; UAS-GFP/+*
